# *In vitro* activity of manogepix and comparators against infrequently encountered yeast and mold isolates from the SENTRY Surveillance Program (2017–2022)

**DOI:** 10.1128/aac.01132-23

**Published:** 2024-01-11

**Authors:** Michael Pfaller, Michael Huband, Paul A. Bien, Cecilia G. Carvalhaes, Abby Klauer, Mariana Castanheira

**Affiliations:** 1 JMI Laboratories, North Liberty, lowa, USA; 2 University of Iowa, Iowa City, lowa, USA; 3 PAB Pharma Consulting LLC, San Diego, California, USA; The University of Iowa, Iowa City, Iowa, USA

**Keywords:** yeasts, molds, manogepix SENTRY surveillance

## Abstract

Manogepix is a potent new antifungal agent targeting the fungal Gwt1 enzyme. Manogepix has previously demonstrated potent *in vitro* activity against clinical isolates of both *Candida* (except *Candida krusei*) and *Aspergillus* species. This study determined the *in vitro* activity of manogepix and comparators against a large collection of infrequently encountered yeast and molds. Manogepix demonstrated potent *in vitro* activity against infrequently encountered yeasts exhibiting elevated MIC values to other drug classes, including *Candida* spp. (MIC_50/90_, 0.008/0.12 mg/L), *Saprochaete clavata* (*Magnusiomyces clavatus*) (MIC_50/90_, 0.03/0.06 mg/L), *Magnusiomyces capitatus* (MIC_range_, 0.016-0.06 mg/L), *Rhodotorula minuta* (MIC, 0.016 mg/L), and *Rhodotorula mucilaginosa* (MIC_50/90_, 0.03/0.12 mg/L). Similarly, manogepix was active against infrequently encountered mold isolates and strains exhibiting elevated MIC/MEC values to echinocandins, azoles, and amphotericin B, including *Coprinopsis cinerea* (MEC, 0.004 mg/L), *Fusarium* spp. (MEC_50/90_, 0.016/0.06 mg/L), *Fusarium* (*Gibberella*) *fujikuroi* species complex (MEC_50/90_, 0.016/0.03 mg/L), *Lomentospora prolificans* (MEC_50/90_, 0.03/0.06 mg/L), *Microascus cirrosus* (MEC, 0.008 mg/L), *Paecilomyces* spp. (MEC_50/90_, ≤0.008/0.016 mg/L), *Pleurostomophora richardsiae* (MEC, 0.06 mg/L), *Sarocladium kiliense* (MEC range, 0.016–0.12 mg/L), and *Scedosporium* spp. (MEC_50/90_, 0.03/0.06 mg/L). Manogepix demonstrated potent activity against a majority of the infrequently encountered yeast and mold isolates tested including strains with elevated MIC/MEC values to other drug classes. Additional clinical development of manogepix (fosmanogepix) in difficult-to-treat, resistant fungal infections is warranted.

## INTRODUCTION

Invasive fungal infections (IFIs) pose a major stumbling block in the case of seriously ill hospitalized individuals (1, 2). Although the majority of IFIs are due to common species of *Aspergillus*, *Candida,* and *Cryptococcus*, less common opportunistic yeasts and molds are emerging as deadly, antifungal-resistant pathogens (3–5).

There are plenty of *in vitro* susceptibility data for common isolates of *Candida albicans, Candida glabrata, Candida parapsilosis, Candida tropicalis,* and *C. krusei* to the available systemically active antifungal agents, amphotericin B, fluconazole, voriconazole, and the echinocandins (6). The emerging, less common species of *Candida* often exhibit multidrug-resistant (MDR; resistant to at least one agent in two or more classes of antifungal agents) profiles (e.g., *Candida auris, Candida guilliermondii, Candida rugosa,* among others) and are a concern that underscores the need for accurate and timely species identification and antifungal susceptibility testing (7–11).

Besides *Candida* and *Cryptococcus* spp., other yeast genera may pose as opportunistic pathogens [e.g., *Saprochaete* (*Magnusiomyces*)*, Rhodotorula, Trichosporon,* and *Saccharomyces* spp.] (7–12). These species often exhibit decreased susceptibility to fluconazole on the order of that typically encountered by fluconazole-resistant strains of *Candida glabrata* and *C. krusei* (13). Cross-resistance between fluconazole and voriconazole may be more prominent for non-*Candida* yeast species than for *Candida* spp. (13). Decreased susceptibility of most non-candidal yeasts to azoles is further complicated by the fact that some species, such as *Saprochaete capitata* (*M. capitatus*)*, S. clavata* (*M. clavatus*)*, Trichosporon asahii,* and *Rhodotorula* spp., are intrinsically resistant to the echinocandins (8, 12, 14).

IFIs due to the filamentous fungi are most often due to *Aspergillus fumigatus* but recently have seen an increase in infections involving non-*fumigatus* species (e.g., *Aspergillus lentulus, Aspergillus ustus* species complex*,* and *Aspergillus udagawae*) and rare molds including the Mucorales, *Fusarium* spp.*, Lomentospora prolificans*, and *Scedosporium* spp. (3, 5, 9, 15–17). These rare molds are known to exhibit highly variable susceptibility to amphotericin B, the echinocandins, and the mold-active azoles (3).

Rare species of fungi are unlikely to be familiar to clinicians and microbiologists alike, and there are few or no data concerning prognosis or optimal treatment strategies. In many instances, these organisms may present as breakthrough infections in immunocompromised hosts who have already been receiving an azole or echinocandin empirically (8, 18).

Besides *Candida* and *A. fumigatus*, few laboratories identify fungi to the species level, and a minority perform antifungal susceptibility testing locally for systemically active antifungal agents (19–21). The identification of emerging, uncommon, yeasts and molds is complicated by the constant change in nomenclature, which may complicate treatment (7, 14). As such, there is a critical role for antifungal surveillance programs that also include the less common fungi. Differences in the regional prevalence and resistance of these fungi render local epidemiological knowledge essential for the care of patients with a suspected IFI (3, 8, 15).

Although Clinical and Laboratory Standards Institute (CLSI) broth microdilution susceptibility testing has been standardized for testing of *Candida* spp., *Cryptococcus* spp.*,* and *Aspergillus* spp. (22–27) and may be applied to rare yeasts and molds, clinical breakpoints and epidemiological cutoff values (ECVs) have not been defined for the rare organisms and any antifungal agent. Regardless, the use of these methods is recommended for the generation of epidemiological data (7, 14). Accurate species identification combined with MIC data may be of use in guiding treatment. Unfortunately, the uncommon nature of these IFIs means that only a single encounter or episode of infection may occur in any given medical center, thus limiting the ability to generate local epidemiological data in a reasonable, clinically relevant time frame. As such, the data generated by comprehensive surveillance programs, such as SENTRY (6, 28), should prove to be a useful reference.

In addressing the antifungal coverage of the rare fungi, it is helpful to understand the activity of conventional antifungal agents as well as that of investigational agents that promise an enhanced spectrum and potency directed at both common and uncommon fungal pathogens (29–31).

Among the recent, systemically active antifungals, manogepix (formerly APX001A and E1210) is noted for its unique mechanism of action. Manogepix targets the highly conserved fungal Gwt1 enzyme (32). Inhibition of Gwt1 blocks the inositol acylation step during the synthesis of glycosylphosphatidylinositol-anchored proteins in the fungal cell wall. Manogepix demonstrates broad-spectrum antifungal activity against common species of *Candida*, *Cryptococcus*, *Aspergillus*, and MDR strains such as *C. auris*, *Trichosporon, Lomentospora*, *Scedosporium*, and rare molds, which are often difficult to treat due to their inherent resistance to many antifungals (28, 32–38).

In this study, we utilized SENTRY Antimicrobial Surveillance Program data from 2017 to 2022 to examine the *in vitro* activity of manogepix and comparators against 1,937 contemporary clinical fungal isolates of rare yeasts and molds from a variety of infections. The isolates were collected from 93 medical centers located in North America (NA), Europe (EU), the Asia-Pacific region (APAC), and Latin America (LA). These data expand on our previous reports from 2017 (36), providing a sizable MIC database for eventual determination of ECVs and clinical breakpoints for manogepix and other antifungal agents and a variety of infrequently encountered fungal species.

## MATERIALS AND METHODS

### Organisms

A total of 1,937 infrequently encountered nonduplicate fungal clinical isolates were selected among 8,512 isolates included in the SENTRY Surveillance Program during 2017–2022 from 93 medical centers located in North America (34 medical centers, 806 isolates), Europe (37 medical centers, 629 isolates), the Asia-Pacific region (15 medical centers, 344 isolates), and Latin America (7 medical centers, 158 isolates). For the purposes of this study, all species of *Candida* less common than *C. krusei*, all *Aspergillus* species less common than *A. fumigatus*, all *Cryptococcus* species, all non-*Candida*, non-*Cryptococcus* yeasts, and all non-*Aspergillus* molds were included. Fungal isolates were recovered from patients with bloodstream infections (630 isolates), respiratory tract infections (543 isolates), skin and skin structure infections (171 isolates), urinary tract infections (35 isolates), intra-abdominal infections (25 isolates), and other infection sites (533 isolates) as determined by local criteria.

### Fungal identification methods

Yeast isolates were subcultured on HardyCHROM agar medium (Hardy Diagnostics, Santa Maria, CA, USA) upon arrival to confirm culture purity for *Candida* spp. isolates and submitted to matrix-assisted laser desorption ionization-time of flight mass spectrometry (MALDI-TOF MS) using the MALDI Biotyper (Bruker Daltonics, Billerica, MA, USA). Any yeast isolates not identified by this process were identified using sequencing-based methods for the internal transcribed spacer (ITS) region, 28S ribosomal DNA, or intergenic spacer 1 for *Trichosporon* spp. (39–42). Mold isolates were identified by DNA sequencing when an acceptable identification was not achieved by MALDI-TOF MS. For all isolates, 28S was sequenced, and one of the following genes was analyzed: β-tubulin for *Aspergillus* spp., translation elongation factor (TEF) for *Fusarium* spp., or ITSs for all other species of filamentous fungi (39–42). Nucleotide sequences were analyzed using Lasergene software (DNAStar, Madison, WI, USA) and compared to available sequences using BLAST. TEF sequences were analyzed using the *Fusarium* multilocus sequence-typing database (https://fusarium.mycobank.org/).

The identification of fungal species (common and uncommon) is complicated by frequent changes in the nomenclature, largely driven by the use of nucleic acid sequence-based identification (43, 44). The adoption of the “one fungus, one name” approach aims to simplify the naming of fungi. In the interest of clinical utility, supporters of nomenclatural stability of medically important fungi have urged caution in adopting new names for fungi (15, 45). In the present survey, we have adopted the approach suggested by previous authors (43, 44) to employ the more familiar name used clinically followed by the newly proposed name in parentheses. This is done the first time it is encountered with the more familiar name used subsequently throughout the manuscript.

### Susceptibility testing

Fungal susceptibility testing was conducted according to broth microdilution methodology as described by CLSI documents M27 (23) and M38 (24). Manogepix MIC and MEC values were determined visually after incubation at 35°C for 24 h (*Candida* spp. MIC) or 48 to 72 h [*Aspergillus* spp. (48 h MEC), other molds (*Scedosporium* spp. 72 h MEC), other yeasts (48 h MIC)]. Yeast MIC endpoints were read as the lowest drug concentration that produced a significant decrease (50% inhibition) of growth below the growth control for manogepix (23, 26, 46), fluconazole, voriconazole, and the echinocandins, or the concentration preventing any discernible growth for amphotericin B (23, 26). Mold MIC endpoints were read as the lowest drug concentration preventing any discernible growth (amphotericin B, itraconazole, and voriconazole) (24, 27). Mold MEC endpoints (morphology change from flocculent growth to small, matted colonies) were read for manogepix and the echinocandins (24, 27, 35). Susceptibility interpretive criteria (clinical breakpoints and ECVs, where available) were those published in CLSI documents M27 (23), M38 (24), M57S (22), M27M44S (26), and M38M51S (27). Resistant (R) breakpoints for *C. auris* and amphotericin B (≥2 mg/L), fluconazole (≥32 mg/L), anidulafungin (≥4 mg/L), and micafungin (≥4 mg/L) were originated from published CDC tentative MIC breakpoints (https://www.cdc.gov/fungal/candida-auris/c-auris-antifungal.html). Tentative statistically derived 97.5% ECVs for *C. auris* and amphotericin B (≤2 mg/L), voriconazole (≤16 mg/L), anidulafungin (≤0.25 mg/L), and micafungin (≤0.25 mg/L) have been published by Arendrup et al. (47).

Clinical breakpoints and ECV values have not yet been determined for manogepix against any fungal species. For comparison, manogepix MIC distributions from the SENTRY Surveillance Program performed in three prior surveys were utilized to generate tentative wild-type upper limits (WT-UL; two twofold dilutions higher than the modal MIC value of each MIC distribution) for several species of *Candida* and *Aspergillus* (28, 36, 37). These WT-UL values were used in the present survey as the cutoff value to define wild-type (MIC ≤ WT Ul) and non-WT (MIC > WT Ul) populations for manogepix and each species for which there were at least 10 isolates (28, 33, 34, 36, 37, 47). The previously published tentative WT-UL values employed in this evaluation were 0.016 mg/L for *Candida dubliniensis,* 0.03 mg/L for *Candida orthopsilosis*, *Aspergillus niger* species complex, and *Aspergillus terreus* species complex, 0.06 mg/L for *Aspergillus flavus* species complex, 0.12 mg/L for *Candida lusitaniae* and *Scedosporium* spp., and 1 mg/L for *Candida kefyr* (28). A tentative 97.5% WT-UL cutoff for manogepix and *C. auris* of 0.03 mg/L have been proposed for the CLSI method by Arendrup et al (34).

Since neither clinical breakpoints nor ECVs are available for most, if not all, of these uncommon organism-antifungal agent combinations, we used the *C. albicans*-resistant clinical breakpoints or ECVs for fluconazole (clinical breakpoint, ≥8 mg/L), voriconazole (clinical breakpoint, ≥1 mg/L), anidulafungin (clinical breakpoint, ≥1 mg/L), micafungin (clinical breakpoint, ≥1 mg/L) and amphotericin B (ECV, ≤1 mg/L) for the rare species of *Candida* and other yeasts and the *A. fumigatus* ECVs for amphotericin B (≤2 mg/L), itraconazole (≤1 mg/L), and voriconazole (≤1 mg/L) for the rare non-*Aspergillus* molds and the non-*fumigatus* species of *Aspergillus* that lack ECVs as a means of identifying species or individual isolates with elevated MIC/MEC ranges (14).

Quality control (QC) was conducted according to CLSI documents M27 (23) and M38 (24) using *Candida parapsilosis* ATCC 22019, *Aspergillus flavus* ATCC 204304, and *Aspergillus fumigatus* ATCC MYA3626. All MIC and MEC values for manogepix against *C. parapsilosis* ATCC 22019, *A. flavus* ATCC 204304, and *A. fumigatus* ATCC MYA-3626 were within QC ranges published in CLSI documents M27M44S (26) and M38M51S (27).

## RESULTS

### Organisms

The frequency distributions and cumulative percent inhibition data for manogepix against the species and organism groups of yeast tested are listed in Tables 1 and 2 and in Tables 3 and 4 for molds. All fungal species containing ≥10 isolates were analyzed separately (Tables 2 and 4). Manogepix and comparator agent susceptibility results for fungal species with fewer than 10 isolates are listed in Tables S1 and S2.

**TABLE 1:** Antifungal activity and cumulative percent inhibition data for manogepix against yeast isolates^*a*^

				Number of occurrences at MIC and cumulative percent inhibition													
Organism (no. tested)	MIC range	MIC_50_	MIC_90_	0.001	0.002	0.004	0.008	0.016	0.03	0.06	0.12	0.25	0.5	1	2	4	>
*Trichosporon mycotoxinivorans* (5) (*Apiotrichum mycotoxinivorans*)	0.5–> 2	>2	–														
*Blastobotrys adeninivorans* (1)	0.004	–	–														
*Candida* spp. (776)	≤0.002–2	0.008	0.12				42855.2	11269.6	9081.2	5588.3	4393.8	2496.9	1498.7	599.4	5100.0		
*C. auris* (77)	≤0.002–0.06	0.016	0.03		45.2	2537.7	949.4	1568.8	1790.9	7100.0							
*C. bracarensis* (4) (*Nakaseomyces bracarensis*)	0.004–0.03	0.008	–														
*C. dubliniensis* (221)	≤0.002–0.03	0.004	0.008		2310.4	13872.9	5899.1	199.5	1100.0								
*C. duobushaemulonii* (4)	≤0.002–0.004	≤0.002															
*C. fabianii* (10) (*Cyberlindnera fabianii*)	≤0.002–0.004	0.004	0.004		440.0	6100.0											
*C. fermentati* (33) (*Meyerozyma caribbica*)	≤0.002–0.06	0.008	0.06		13.0	724.2	1157.6	778.8	284.8	5100.0							
*C. guilliermondii* (27) (*Meyerozyma guilliermondii*)	0.004–0.016	0.008	0.016		00.0	725.9	959.3	11100.0									
*C. haemulonii* (6)	≤0.002–0.004	≤0.002	–														
*C. inconspicua* (6) (*Pichia cactophila*)	0.5–2	2	–														
*C. intermedia* (2)	0.004–0.03	0.004	–														
*C. kefyr* (78)	0.03–1	0.12	0.5					00.0	45.1	1220.5	3160.3	2187.2	897.4	2100			
*C. krusei* (202)	0.25–>2	>2	>2								00.0	21.0	32.5	02.5	34.0		194100.0
*C. lipolytica* (7) (*Yarrowia lipolytica*)	0.006–0.06	0.03	–														
*C. lusitaniae* (150) (*Clavispora lusitaniae*)	0.004–0.5	0.03	0.06		00.0	21.3	65.3	4736.7	5271.3	2890.0	1197.3	298.7	2100.0				
*C. metapsilosis* (34)	0.004–0.03	0.008	0.008		00.0	1544.1	1691.2	297.1	1100.0								
*C. nivariensis* (6) (*Nakaseomyces nivariensis*)	≤0.002–0.008	0.004	–														
*C. norvegensis* (7) (*Pichia norvegensis*)	0.12–1	0.5	–														
*C. orthopsilosis* (66)	0.004–0.03	0.008	0.016		00.0	1218.2	2454.5	2490.9	6100.0								
*C. pararugosa* (3) (*Diutina pararugosa*)	≤0.002	≤0.002	–														
*C. pelliculosa* (13) (*Wickerhamomyces anomalus*)	≤0.002	≤0.002	≤0.002		13100.0												
*C. pseudohaemulonii* (2)	0.004	0.004	–														
*C. quercitrusa* (1)	0.016	–	–														
*C. rugosa* (5) (*Diutina rugosa*)	0.004–0.03	0.016	–														
*C. spencermartinsiae* (1)	0.008	–	–														
*C. sphaerica* (3)	0.06–0.25	0.06	–														
*C. theae* (2)	0.004	0.004	–														
*C. utilis* (7)	≤0.002–0.008	≤0.002	–														
*Cryptococcus gattii* species complex (6)	0.12–2	0.25	–														
*C. laurentii* (1) (*Papiliotrema laurentii*)	0.25	–	–														
*C. neoformans* (178) (*C. neoformans* var. *grubii*)	0.016–4	0.25	1				00.0	21.1	149.0	2422.5	4648.3	2461.8	3682.0	1892.1	1198.3	3100.0	
*C. deneoformans* (13) (*C. neoformans* var. *neoformans*)	0.03–1	0.25	0.5					00.0	215.4	015.4	123.1	453.8	592.3	1100.0			
*Hyphopichia burtonii* (1)	0.001																
*Kodamaea ohmeri* (4)	0.008–0.016	0.008	–														
*Lodderomyces elongisporus* (1)	0.004	–	–														
*Saprochaete* (*Magnusiomyces*) *capitatus* (4)	0.016–0.06	0.016	–														
*Saprochaete* (*Magnusiomyces*) *clavatus* (14)	0.016–0.06	0.03	0.06		00.0	18.3	983.3	2100.0									
*Ogataea siamensis* (1)	0.03	–	–														
*Pichia cactophila* (2)	0.5–1	0.5	–														
*P. kluyveri* (1)	0.06	–	–														
*Rhodotorula minuta* (1)	0.016	–	–														
*R. mucilaginosa* (18)	0.016–0.5	0.03	0.12				00.0	211.1	961.1	588.9	194.4	094.4	1100.0				
*Saccharomyces cerevisiae* (28)	0.008–0.06	0.016	0.03			00.0	932.1	1067.9	896.4	1100.0							
*Trichosporon asahii* (26)	0.25–> 2	>2	>2								00.0	311.5	426.9	334.6	346.2		14100.0
*T. capitatum* (1)	0.03	–	–														
*T. inkin* (2)	1–2	1	–														
*T. loubieri* (1) (*Apiotrichum loubieri*)	0.5	–	–														
*T. mucoides* (2) (*Cutaneotrichosporon mucoides*)	>2	>2	–														

^*a*^ –, no value.

**TABLE 2:** *In vitro* activity of manogepix and comparators against infrequently encountered yeast isolates from the SENTRY Surveillance Program (2017–2022)

		MIC (mg/L)			No. and cumulative percent of isolates inhibited at MIC (mg/L) of:										
Organism (no. tested)	Antifungal agent	MIC range	MIC_50_	MIC_90_	≤0.008	0.016	0.03	0.06	0.12	0.25	0.5	1	2	4	≥8
*Candida* spp. (776)^*a*^	Manogepix	≤0.002–2	0.008	0.12	42855.2	11269.6	9081.2	5588.3	4393.8	2496.9	1498.7	599.4	5100.0		
	Fluconazole	≤0.12–> 128	0.5	64					22028.4	15948.8	11463.5	5971.1	6078.9	3983.9	125100.0
	Voriconazole	≤0.008–8	≤0.008	0.25	41153.0	11267.4	5774.7	4780.8	5287.5	2690.9	1893.2	3297.3	1499.1	599.7	2100.0
	Anidulafungin	≤0.002–4	0.12	1	101.3	447.0	12222.7	13339.8	8851.2	15871.5	13588.9	5195.5	3399.7	2100.0	
	Micafungin	≤0.008–4	0.12	0.5	162.1	13819.8	15239.4	7449.0	16169.7	14388.1	7597.8	1699.9	099.9	1100.0	
	Amphotericin B	≤0.12–4	0.5	1				00.0	293.7	22733.0	31773.8	18697.8	1699.9	1100.0	
*C. auris* (77)	Manogepix	≤0.002–0.06	0.016	0.03	949.9	1568.8	1790.9	7100.0							
	Fluconazole	2–>128	128	>128								00.0	22.6	610.4	69100.0
	Voriconazole	0.016–4	1	2	00.0	11.3	46.5	513.0	823.4	1745.5	248.1	2783.1	1298.7	1100.0	
	Anidulafungin	0.12–4	0.25	0.5				00.0	45.2	4057.1	2994.8	398.7	098.7	1100.0	
	Micafungin	0.03–4	0.25	0.25			00.0	56.5	3344.9	3898.7	098.7	098.7	098.7	1100.0	
	Amphotericin B	0.5–4	1	2						00.0	911.7	5785.7	1098.7	1100.0	
*C. dubliniensis* (221)	Manogepix	≤0.002–0.03	0.004	0.008	21999.1	199.5	1100.0								
	Fluconazole	≤0.12–64	≤0.12	0.25					16976.5	4898.2	198.6	199.1	199.5	099.5	1100.0
	Voriconazole	≤0.008–0.12	≤0.008	≤0.008	19990.0	1898.2	299.1	199.5	1100.0						
	Anidulafungin	0.004–0.5			4 1.8	2211.8	8851.6	7786.4	2899.1	099.1	2100.0				
	Micafungin	0.008–1			115.0	12059.3	8296.4	699.1	099.1	099.1	099.1	2100.0			
	Amphotericin B	0.12–1			5899.1	199.5	1100.0								
*C. fabianii* (10) (*Cyberlindnera fabianii*)	Manogepix	≤0.002–0.004	0.004	0.004	10100.0										
	Fluconazole	0.25–4	1	2					00.0	110.0	010.0	450.0	490.0	1100.0	
	Voriconazole	0.008–0.06	0.03	0.06	110	120.0	680.0	2100.0							
	Anidulafungin	0.03–1	0.06	0.06		00.0	330.0	690.0	090.0	090.0	090.0	1100.0			
	Micafungin	0.016–0.5	0.03	0.03	00.0	220.0	790.0	090.0	090.0	090.0	090.0	1100.0			
	Amphotericin B	0.25–1	0.5	1					00.0	110.0	780.0	2100.0			
*C. fermentati* (33) (*Meyerozyma caribbica*)	Manogepix	≤0.002–0.06	0.008	0.06	1957.6	778.8	2 84.8	5100.0							
	Fluconazole	0.25–64	2	32					00.0	13.0	03.0	518.2	1151.5	669.7	10100.0
	Voriconazole	0.03–1	0.12	0.5		00.0	13.0	1342.4	1175.8	384.8	290.9	3100.0			
	Anidulafungin	0.12–2	1	2				00.0	13.0	415.2	839.4	1687.9	4100.0		
	Micafungin	0.12–1	0.25	0.5				00.0	927.3	1572.7	897.0	1100.0			
	Amphotericin B	0.5–1	0.5	1						00.0	2266.7	11100.0			
*C. guilliermondii* (27) (*Meyerozyma guilliermondii*)	Manogepix	0.004–0.016	0.008	0.016	1659.3	11100.0									
	Fluconazole	0.03–>128	2	>128		00.0	13.7	03.7	03.7	03.7	03.7	03.7	1351.9	570.4	8100.0
	Voriconazole	0.008–8	0.06	4	13.7	03.7	729.6	963.0	477.8	077.8	077.8	181.5	785.2	396.3	1100.0
	Anidulafungin	1–4	2	2							00.0	622.2	2096.3	1100.0	
	Micafungin	0.12–1	0.5	1				00.0	13.7	211.1	1981.5	5100.0			
	Amphotericin B	0.25–1	0.5	0.5					00.0	518.5	2092.6	2100.0			
*C. kefyr* (78)	Manogepix	0.03–1	0.12	0.5		00.0	45.1	1220.5	3160.3	2187.2	897.4	2100.0			
	Fluconazole	≤0.12–1	0.25	0.5				00.0	2430.8	2866.7	2294.9	4100.0			
	Voriconazole	≤0.008–0.03	≤0.008	0.016	6178.2	1597.4	2100.0								
	Anidulafungin	0.016–0.25	0.06	0.12	00.0	56.4	1728.2	3573.1	1896.2	3100.0					
	Micafungin	0.016–0.12	0.03	0.06	00.0	11.3	4052.6	3192.3	6100.0						
	Amphotericin B	0.12–2	1	1				00.0	11.3	01.3	1419.2	6298.7	1100.0		
*C. lusitaniae* (150) (*Clavispora lusitaniae*)	Manogepix	0.004–0.5	0.03	0.06	85.3	4736.7	5271.3	2890.0	1197.3	298.7	2100.0				
	Fluconazole	≤0.12–64	0.25	2				00.0	2516.7	6157.3	3782.0	1189.3	492.0	293.3	10100.0
	Voriconazole	≤0.008–0.5	≤0.008	0.016	12080.0	1791.3	393.3	194.0	295.3	296.7	5100.0				
	Anidulafungin	0.12–1	0.5	0.5			00.0	21.3	1410.7	7359.3	5596.0	6100.0			
	Micafungin	0.03–0.5	0.12	0.25		00.0	21.3	1511.3	9876.7	3197.3	4100.0				
	Amphotericin B	0.12–1	0.5	0.5				00.0	21.3	5034.7	8490.7	14100.0			
*C. metapsilosis* (34)	Manogepix	0.004–0.03	0.008	0.008	3191.2	297.1	1100.0								
	Fluconazole	0.25–8	1	2					00.0	25.9	1344.1	1279.4	491.2	194.1	2100.0
	Voriconazole	≤0.008–0.12	0.016	0.03	617.6	1870.6	791.2	297.1	1100.0						
	Anidulafungin	0.06–0.5	0.25	0.5			00.0	12.9	1135.3	1888.2	4100.0				
	Micafungin	0.12–1	0.25	0.5				00.0	514.7	1970.6	997.1	1100.0			
	Amphotericin B	0.25–1	0.5	0.5					00.0	38.8	2891.2	3100.0			
*C. orthopsilosis* (66)	Manogepix	0.004–0.03	0.008	0.016	3654.5	2490.9	6100.0								
	Fluconazole	0.12–>128	0.5	4				00.0	11.5	1727.3	3275.8	887.9	087.9	290.9	6100.0
	Voriconazole	≤0.008–8	0.016	0.12	1725.8	2969.7	1084.8	287.9	290.9	090.9	497.0	097.0	097.0	097.0	2100.0
	Anidulafungin	0.12–2	0.5	2				00.0	23.0	1221.2	2863.6	1789.4	7100.0		
	Micafungin	0.12–1	0.25	0.5				00.0	11.5	3351.5	2792.4	5100.0			
	Amphotericin B	0.12–1	0.5	0.5				00.0	23.0	1627.3	4697.0	2100.0			
*C. pelliculosa* (13) (*Wickerhamomyces anomalus*)	Manogepix	≤0.002	≤0.002	≤0.002	13100.0										
	Fluconazole	1–64	2	4							00.0	17.7	869.2	392.3	1100.0
	Voriconazole	0.06–2	0.12	0.25			00.0	430.8	784.6	192.3	092.3	092.3	1100.0		
	Anidulafungin	≤0.002–0.03	0.016	0.03	430.8	784.6	2100.0								
	Micafungin	≤0.002–0.06	0.03	0.06	17.7	223.1	884.6	2100.0							
	Amphotericin B	0.25–1	0.5	1					00.0	538.5	576.9	3100.0			
*Cryptococcus* spp. (199)^*b*^	Manogepix	0.016–4	0.25	1	00.0	21.0	169.0	2421.1	4845.2	3160.8	4382.4	2092.5	1298.5	3100.0	
	Fluconazole	0.5–16	2	4						00.0	84.1	2315.9	7453.8	7290.8	18100.0
	Voriconazole	0.008–0.25	0.03	0.06	00.0	42.1	1911.8	9259.0	6391.3	1599.0	2100.0				
	Amphotericin B	≤0.12–1	0.5	1				00.0	21.0	179.7	11970.8	57100.0			
*C. neoformans* (178) (*C. neoformans* var. *grubii*)	Manogepix	0.016–4	0.25	1	00.0	21.1	149.0	2422.5	4648.3	2461.8	3682.0	1892.1	1198.3	3100.0	
	Fluconazole	0.5–16	2	4						00.0	52.9	1712.6	7052.9	6590.2	17100.0
	Voriconazole	0.008–0.25	0.03	0.06	42.3	1510.9	8559.8	5792.5	1299.4	1100.0					
	Amphotericin B	0.25–1	0.5	1					00.0	105.7	10968.4	55100.0			
*C. deneoformans* (13) (*C. neoformans* var. *neoformans*)	Manogepix	0.03–1	0.25	0.5		00.0	215.4	123.1	453.8	592.3	1100.0				
	Fluconazole	0.5–4	1	4						00.0	323.1	669.2	284.6	2100.0	
	Voriconazole	0.016–0.06	0.03	0.06	00.0	430.8	676.9	3100.0							
	Amphotericin B	≤0.12–0.5	0.25	0.5				00.0	215.4	553.6	6100.0				
*Saprochaete* (*Magnusiomyces*) spp. (16)^*c*^	Manogepix	0.016–0.06	0.03	0.06	00.0	318.8	1081.2	3100.0							
	Fluconazole	1–16	8	16							00.0	16.2	06.2	218.8	13100.0
	Voriconazole	0.06–0.5	0.12	0.25			00.0	212.5	756.2	693.8	1100.0				
	Anidulafungin	2–4	4	4								00.0	637.5	10100.0	
	Micafungin	1–>4	>4	>4							00.0	425.0	237.5	143.8	9100.0
	Amphotericin B	0.5–1	1	1						00.0	16.2	15100.0			
*Saprochaete clavata* (12) (*Magnusiomyces clavatus*)	Manogepix	0.016–0.06	0.03	0.06	00.0	18.3	983.3	2100.0							
	Fluconazole	4–16	8	16									00.0	216.7	10100.0
	Voriconazole	0.06–0.5	0.12	0.25			00.0	18.3	658.3	491.7	1100.0				
	Anidulafungin	2–4	4	4								00.0	541.7	7100.0	
	Micafungin	1–>4	>4	>4								00.0	433.3	141.7	7100.0
	Amphotericin B	0.5–1	1	1						00.0	18.3	11100.0			
*Rhodotorula mucilaginosa* (18)	Manogepix	0.016–0.5	0.03	0.12	00.0	211.1	961.1	588.9	194.4	094.4	1100.0				
	Fluconazole	0.5–>128	128	>128							15.6	05.6	05.6	05.6	17100.0
	Voriconazole	0.03–4	0.5	2		00.0	15.6	05.6	111.1	222.2	655.6	477.8	394.4	1100.0	
	Anidulafungin	>4	>4	>4										00.0	18100.0
	Micafungin	>4	>4	>4										00.0	18100.0
	Amphotericin B	0.25–1	0.5	0.5					00.0	15.6	1694.4	1100.0			
*Saccharomyces cerevisiae* (28)	Manogepix	0.008–0.06	0.016	0.03	932.1	1067.9	896.4	1100.0							
	Fluconazole	0.5–32	2	16						00.0	310.7	321.4	953.6	675.0	7100.0
	Voriconazole	0.008–0.5	0.06	0.25	13.6	17.1	835.7	864.3	685.7	292.9	2100.0				
	Anidulafungin	0.03–1	0.12	0.5		00.0	27.1	421.4	1057.1	782.1	496.4	1100.0			
	Micafungin	0.06–0.25	0.12	0.25			00.0	517.9	1571.4	8100.0					
	Amphotericin B	0.25–1	0.5	1					00.0	414.3	1567.9	9100.0			
*Trichosporon* spp. (33)^*d*^	Manogepix	0.03–>2	>2	>2		00.0	13.0	03.0	03.0	312.1	527.2	336.4	448.5	048.5	17100.0
	Fluconazole	0.25–>128	4	64					00.0	13.0	312.1	424.2	436.4	1066.7	11100.0
	Voriconazole	0.008–>8	0.06	1	26.1	212.1	630.3	1060.6	472.7	484.8	187.9	190.9	297.0	097.0	1100.0
	Anidulafungin	2–>4	>4	>4								00.0	13.0	312.1	29100.0
	Micafungin	2–>4	>4	>4								00.0	13.0	03.0	32100.0
	Amphotericin B	0.5–2	1	1						00.0	824.2	2497.0	1100.0		
*T. asahii* (26)	Manogepix	0.25–>2	>2	>2					00.0	311.5	426.9	234.6	346.2	046.2	14100.0
	Fluconazole	1–128	4	64							00.0	415.4	430.8	965.4	9100.0
	Voriconazole	0.016–2	0.06	1	00.0	13.8	626.9	857.7	473.1	384.6	188.5	192.3	2100.0		
	Anidulafungin	4–>4	>4	>4									00.0	27.7	24100.0
	Micafungin	>4	>4	>4										0.0	26100.0
	Amphotericin B	0.5–2	1	1						00.0	415.4	2196.2	1100.0		

^*a*^ Contains *Candida auris* (77), *C. bracarensis* (4), *C. dubliniensis* (221), *C. duobushaemulonii* (4), *C. fabianii* (10), *C. fermentati* (33), *C. guilliermondii* (27), *C. haemulonii* (6), *C. intermedia* (2), *C. kefyr* (78), *C. lipolytica* (7), *C. lusitaniae* (150), *C. metapsilosis* (34), *C. nivariensis* (6), *C. norvegensis* (7), *C. orthopsilosis* (66), *C. pararugosa* (3), *C. pelliculosa* (13), *C. pseudohaemulonii* (2), *C. quercitrusa* (1), *C. rugosa* (5), *C. spencermartinsiae* (1), *C. sphaerica* (3), *C. theae* (2), *C. utilis* (7), and unspeciated *Candida* (1).

^*b*^ Contains *Cryptococcus gattii* species complex (6), *C. laurentii* (1), *C. neoformans* (178), and *C. deneoformans* (13).

^*c*^ Contains *Saprochaete capitata* (4) and *S. clavata* (12).

^*d*^ Contains *Trichosporon asahii* (26), *T. capitatum* (1), *T. inkin* (2), *T. mucoides* (2), and unspeciated *Trichosporon* (1).

**TABLE 3:** Antifungal activity and cumulative percent inhibition data for manogepix against mold isolates^*a*^

				Number of occurrences at MIC and cumulative percent inhibition													
Organism (no. tested)	MIC range	MIC_50_	MIC_90_	0.001	0.002	0.004	0.008	0.016	0.03	0.06	0.12	0.25	0.5	1	2	4	>
*Alternaria alternata* (1)	1	–	–														
*Aspergillus alabamensis* (1)	0.008	–	–														
*A. brasiliensis* (2)	0.008–0.016	0.008	–														
*A. clavatus* (2)	0.03	0.03	–														
*A. flavus* species complex (173)	0.004–0.12	0.016	0.06			00.0	3017.3	5850.9	6085.5	2398.8	2100.0						
*A. fumisynnematus* (1)	0.008	–	–														
*A. hortai* (1)	0.008	–	–														
*A. lentulus* (7)	0.008–0.016	0.008	–														
*A. melleus* (1)	0.016	–	–														
*A. nidulans* species complex (42)	0.008–0.03	0.016	0.03			00.0	819.0	2066.7	14100.0								
*A. niger* species complex (185)	≤0.008–0.12	≤0.008	0.016				12064.9	4790.3	1095.7	799.5	1100.0						
*A. nomius* (1)	0.008	–	–														
*A. ochraceus* species complex (1)	0.12	–	–														
*A. parasiticus* (3)	0.008–0.016	0.008	–														
*A. sclerotiorum* (3)	0.016–0.03	0.016	–														
*A. sydowii* (4)	0.002–0.016	0.016	–														
*A. tamarii* (3)	0.03–0.06	0.03	–														
*A. terreus* species complex (71)	0.004–0.03	0.016	0.03		00.0	22.8	2436.6	3484.5	11100.0								
*A. thermomutatus* (2)	0.06–0.25	0.06	–														
*A. tubingensis* (23)	≤0.008–0.03	≤0.008	0.03				1773.9	282.6	4100.0								
*A. udagawae* (2)	0.016	0.016	–														
*A. unguis* (3)	0.03	0.03	–														
*A. ustus* species complex (12)	≤0.008–0.016	≤0.008	0.016				1083.3	2100.0									
*A. versicolor* (7)	≤0.002–0.03	0.016	–														
*A. welwitschiae* (1)	0.016	–	–														
*Aureobasidium pullulans* (2)	0.008	–	–														
*Coprinopsis cinerea* (1)	0.004	–	–														
*Exophiala attenuata* (2)	0.008–0.016	0.008	–														
*E. dermatitidis* (10)	≤0.008	≤0.008	≤0.008				10100.0										
*Fusarium annalatum* (2)	0.008–0.03	0.008	–														
*F. dimerum* species complex (1)	0.06	–	–														
*F. falciforme* (1)	0.016	–	–														
*F. incarnatum-equiseti* species complex (4)	≤0.002–8	0.12	–														
*F. oxysporum* species complex (8)	0.008–4	0.03	–														
*F. petroliphilum* (1)	0.016	–	–														
*F. solani* species complex (29)	0.004–0.03	0.016	0.03		00.0	26.9	317.2	1775.9	7100.0								
*F*. (*Gibberella*) *fujikuroi* species complex (21)	≤0.008–0.12	0.016	0.03				838.1	876.2	390.5	090.5	2100						
*Lichtheimia corymbifera* (8)	4–>4	>4	–														
*L. ramosa* (1)	>4	–	–														
*Lomentospora prolificans* (19)	0.004–0.06	0.03	0.06		00.0	15.3	215.8	436.8	773.7	5100.0							
*Medicopsis romeroi* (2)	0.03–0.12	0.03	–														
*Microascus cirrosus* (1)	0.008	–	–														
*Monascus ruber* (1)	0.03	–	–														
*Mucor circinelloides* (8)	0.25–>4	2	–														
*M. circinelloides/M. ramosissimus* (2)	1–2	1	–														
*M. indicus* (1)	1	–	–														
*Paecilomyces variotii* (10)	≤0.008	≤0.008	≤0.008				10100.0										
*Penicillium citrinum* (1)	0.008	–	–														
*P. onobense* (1)	0.008	–	–														
*Pleurostomophora parasiticum* (1)	0.06	–	–														
*P. richardsiae* (1)	0.06	–	–														
*Pseudopithomyces sacchari* (1)	0.25	–	–														
*Purpureocillium lilacinum (Paecilomyces lilacinus)* (15)	≤0.008–0.016	≤0.008	0.016				1173.3	4100.0									
*Rasamsonia argillacea* species complex (12)	≤0.008–0.016	≤0.008	0.016				866.7	4100.0									
*Rhizomucor pusillus* (6)	1–>4	4	–														
*Rhizopus microsporus* group (24)	2–> 4	4	>4											00.0	312.5	1262.5	9100.0
*Rhizopus oryzae* species complex (19)	0.5–>4	>4	>4									00.0	15.3	05.3	05.3	426.3	14100.0
*Sarocladium kiliense* (4)	0.016–0.12	0.03	–														
*Scedosporium apiospermum/S. boydii* (58)	0.004–0.5	0.03	0.06		00.0	23.4	512.1	1639.7	2379.3	994.8	298.3	098.3	1100.0				
*S. aurantiacum* (11)	0.016–0.06	0.03	0.06				00.0	318.2	563.6	4100.0							
*S. dehoogi* (3)	0.03–0.06	0.03	–														
*S. minutisporum* (2)	0.008–0.016	0.008	–														
*Scopulariopsis brevicaulis*/*S. brumptii* (3)	≤0.002–0.008	0.008															
*Trichoderma longibrachiatum* (1)	0.06	–	–														
*Verruconis gallopava* (2)	0.5–1	0.5	–														

^*a*^ –, no value.

**TABLE 4:** *In vitro* activity of manogepix and comparators against infrequently encountered mold isolates from the SENTRY Surveillance Program (2017–2022)^*m*^

		MIC (mg/L)			No. and cumulative percent of isolates inhibited at MIC (mg/L) of:										
Organism (no. tested)	Antifungal agent	MIC range	MIC_50_	MIC_90_	≤0.008	0.016	0.03	0.06	0.12	0.25	0.5	1	2	4	≥8
*Aspergillus* spp. (548)^*a*^	Manogepix	≤0.008–0.25	0.016	0.03	22240.5	18173.5	10893.2	3299.1	499.8	1100.0					
	Anidulafungin	≤0.008–0.5	≤0.008	0.03	35864.5	11987.2	4896.0	1598.7	699.8	099.8	1100.0				
	Micafungin	≤0.008–0.5	≤0.008	0.03	30856.3	17888.8	5398.5	599.5	299.8	1100.0					
	Itraconazole	0.12–>8	1	4				00.0	81.5	4710.1	18644.1	14370.3	8585.9	3592.3	42100.0
	Voriconazole	0.03–8	0.5	2		00.0	20.4	112.4	318.0	7621.9	19457.3	14483.6	6896.0	1698.9	6100.0
	Amphotericin B	0.06–>4	1	2			00.0	50.9	336.9	4615.3	11836.9	14763.7	17796.0	1799.1	5100.0
*A. flavus* species complex (173)^*b*^	Manogepix	≤0.008–0.12	0.016	0.06	3017.3	5850.9	6085.5	2398.8	2100.0						
	Anidulafungin	≤0.008–0.03	≤0.008	0.016	12974.6	3896.5	6100.0								
	Micafungin	≤0.008–0.03	0.016	0.03	6537.6	8787.9	21100.0								
	Itraconazole	0.12–2	0.5	1				00.0	10.6	1710.5	10973.8	4499.4	1100.0		
	Voriconazole	0.25–2	0.5	1					00.0	3017.3	8465.9	5597.7	4100.0		
	Amphotericin B	0.12–>4	2	2				00.0	10.6	21.7	1611.0	6347.4	8496.0	699.4	1100.0
*A. nidulans* species complex (42)^*c*^	Manogepix	0.008–0.03	0.016	0.03	819.0	2066.7	14100.0								
	Anidulafungin	≤0.008–0.12	0.016	0.06	1023.8	1354.8	1385.7	495.2	2100.0						
	Micafungin	≤0.008–0.25	≤0.008	0.03	2150.0	1278.6	795.2	197.6	097.6	1100.0					
	Itraconazole	0.12–1	0.5	1				00.0	37.1	519.0	2373.8	11100.0			
	Voriconazole	0.03–0.25	0.12	0.25		00.0	24.8	721.4	2273.8	995.2	2100.0				
	Amphotericin B	0.12–>4	2	2				00.0	24.8	04.8	619.0	735.7	2697.6	097.6	1100.0
*A. niger* species complex (185)^*d*^	Manogepix	≤0.008–0.12	≤0.008	0.016	12064.9	4790.3	1095.7	799.5	1100.0						
	Anidulafungin	≤0.008–0.03	≤0.008	0.016	15784.9	2598.4	3100.0								
	Micafungin	≤0.008–0.06	≤0.008	0.016	12869.2	4291.9	1398.9	2100.0							
	Itraconazole	0.12–>8	2	4				00.0	21.1	11.6	64.9	6339.1	7177.7	2591.3	16100.0
	Voriconazole	0.06–4	1	2			00.0	10.5	11.1	32.7	6537.8	7779.5	3598.4	3100.0	
	Amphotericin B	0.06–2	0.5	1			00.0	21.1	1710.3	3931.4	8275.7	4197.8	4100.0		
*A. terreus* species complex (71)^*e*^	Manogepix	0.004–0.03	0.016	0.03	2636.6	3484.5	11100.0								
	Anidulafungin	≤0.002–0.06	0.016	0.03	2839.4	2676.1	1597.2	2100.0							
	Micafungin	≤0.008–0.016	≤0.008	0.016	5577.5	16100.0									
	Itraconazole	0.12–1	0.5	1				00.0	22.8	2132.4	3885.9	10100.0			
	Voriconazole	0.06–2	0.5	0.5			00.0	22.8	611.3	2749.3	3091.5	598.6	1100.0		
	Amphotericin B	0.25–4	2	2					00.0	11.4	711.3	1633.8	4090.1	7100.0	
*A. tubingensis* (23)	Manogepix	≤0.008–0.03	≤0.008	0.03	1773.9	282.6	4100.0								
	Anidulafungin	≤0.008–0.03	≤0.008	0.03	1878.3	495.7	1100.0								
	Micafungin	≤0.008–0.03	≤0.008	0.03	1356.5	682.6	4100.0								
	Itraconazole	2–>8	8	>8								00.0	417.4	643.5	13100.0
	Voriconazole	1–4	2	2							00.0	14.3	1987.0	3100.0	
	Amphotericin B	0.06–1	0.12	0.5			00.0	28.7	1156.5	369.6	591.3	2100.0			
*A. ustus* species complex (12)^*f*^	Manogepix	≤0.008–0.006	≤0.008	0.016	1083.3	2100.0									
	Anidulafungin	≤0.008–0.12	0.016	0.12	541.7	150.0	375.0	183.3	2100.0						
	Micafungin	≤0.008–0.06	0.016	0.03	433.3	683.3	191.7	1100.0							
	Itraconazole	0.25–>8	8	>8					00.0	18.3	08.3	116.7	225.0	350.0	6100.0
	Voriconazole	0.25–8	4	8					00.0	18.3	08.3	08.3	08.3	658.3	5100.0
	Amphotericin B	0.25–4	1	4					00.0	18.3	116.7	666.7	283.3	2100.0	
*Exophiala dermatitidis* (10)	Manogepix	≤0.008	≤0.008	≤0.008	10100.0										
	Anidulafungin	0.12–>4	>4	>4				00.0	110.0	010.0	230.0	030.0	030.0	030.0	7100.0
	Micafungin (*n* = 9)	0.016–>4	>4	–	00.0	111.1	011.1	122.2	133.3	033.3	033.3	033.3	033.3	033.3	6100.0
	Itraconazole	0.12–0.5	0.5	0.5				00.0	110.0	120.0	8100.0				
	Voriconazole	0.06–0.5	0.12	0.25			00.0	330.0	250.0	490.0	1100.0				
	Amphotericin B	0.06–1	0.5	1			00.0	110.0	120.0	130.0	360.0	4100.0			
*Fusarium* spp. (67)^*g*^	Manogepix	≤0.008–8	0.016	0.06	1623.9	2967.2	1285.1	491.0	395.5	095.5	095.5	095.5	197.0	198.5	1100.0
	Anidulafungin	0.004–>4	>4	>4	23.0	03.0	03.0	03.0	03.0	03.0	03.0	03.0	03.0	14.5	64100.0
	Micafungin	0.004–>4	>4	>4	23.0	03.0	03.0	03.0	03.0	03.0	03.0	03.0	26.0	310.4	60100.0
	Itraconazole	2–>8	>8	>8								00.0	11.5	01.5	66100.0
	Voriconazole	2–>8	8	>8								00.0	710.4	1837.3	42100.0
	Amphotericin B	0.12–>2	2	>2				00.0	23.0	37.5	311.9	2041.8	3289.6	089.6	7100.0
*F. solani* species complex (29)^*h*^	Manogepix	0.004–0.03	0.016	0.03	517.2	1775.9	7100.0								
	Anidulafungin	0.004–>4	>4	>4	13.4	03.4	03.4	03.4	03.4	03.4	0 3.4	03.4	03.4	03.4	28100.0
	Micafungin	0.008–>4	>4	>4	13.4	03.4	03.4	03.4	03.4	03.4	03.4	03.4	03.4	313.8	25100.0
	Itraconazole	>8 >8	>8												29100.0
	Voriconazole	4–>8	8	>8									00.0	620.7	23100.0
	Amphotericin B	0.12–4	1	2				00.0	26.9	110.3	010.3	1251.7	1293.1	2100.0	
*F*. (*Gibberella*) *fujikuroi* species complex (21)	Manogepix	≤0.008–0.12	0.016	0.03	838.1	876.2	390.5	090.5	1100.0						
	Anidulafungin	0.008–>4	>4	>4	14.8	04.8	04.8	04.8	04.8	04.8	04.8	04.8	04.8	04.8	20100.0
	Micafungin	0.004–>4	>4	>4	14.8	04.8	04.8	04.8	04.8	04.8	04.8	04.8	04.8	04.8	20100.0
	Itraconazole	8–>8	>8	>8										00.0	21100.0
	Voriconazole	2–>8	4	>8								00.0	314.3	852.4	10100.0
	Amphotericin B	0.25–>2	2	>2					00.0	29.5	219.0	123.8	1281.0	091.0	4100.0
*Lomentospora prolificans* (19)	Manogepix	0.004–0.06	0.03	0.06	315.8	436.8	773.7	5100.0							
	Anidulafungin	2–>4	>4	>4								00.0	15.3	847.4	10100.0
	Micafungin	>4	>4	>4										00.0	19100.0
	Itraconazole	>8	>8	>8										00.0	19100.0
	Voriconazole	>8	>8	>8										00.0	19100.0
	Amphotericin B	2–>2	>2	>2								00.0	15.3	00.0	18100.0
*Mucorales* (69)^*i*^	Manogepix	0.25–>4	>4	>4					00.0	22.9	14.3	410.1	517.4	2147.8	36100.0
	Anidulafungin	4–>4	>4	>4									00.0	811.6	61100.0
	Micafungin	>4	>4	>4										00.0	69100.0
	Itraconazole	0.5–>8	2	8						00.0	57.2	2340.6	1866.7	873.8	15100.0
	Voriconazole	4–>8	>8	>8									00.0	34.3	66100.0
	Amphotericin B	0.06–2	0.5	1			00.0	68.7	718.8	931.9	2669.6	2098.6	1100.0		
*Mucor* spp. (11)^*j*^	Manogepix	0.25–>4	2	>4					00.0	218.2	018.2	345.5	263.6	172.7	3100.0
	Anidulafungin	4–>4	>4	>4									00.0	218.2	9100.0
	Micafungin	>4	>4	>4										00.0	11100.0
	Itraconazole	1–>8	8	>8							00.0	19.1	336.4	145.5	6100.0
	Voriconazole	>8	>8	>8											11100.0
	Amphotericin B	0.06–0.5	0.25	0.5			00.0	218.2	018.2	563.6	4100.0				
*Rhizopus microsporus* group (24)	Manogepix	2–>4	4	>4								00.0	312.5	1262.5	9100.0
	Anidulafungin	>4	>4	>4										00.0	24100.0
	Micafungin	>4	>4	>4										00.0	24100.0
	Itraconazole	1–>8	2	>8							00.0	625.0	962.5	375.0	6100.0
	Voriconazole	8–>8	8	>8										00.0	24100.0
	Amphotericin B	0.06–2	0.5	1			00.0	14.2	316.7	225.0	650.0	1195.8	1100.0		
*R. oryzae* species complex (19)	Manogepix	0.5–>4	>4	>4						00.0	15.3	05.3	05.3	426.3	14100.0
	Anidulafungin	>4	>4	>4										00.0	19100.0
	Micafungin	>4	>4	>4										00.0	19100.0
	Itraconazole	0.5–8	1	8						00.0	315.8	752.6	473.7	284.2	3100.0
	Voriconazole	4–>8	8	>8									00.0	315.8	16100.0
	Amphotericin B	0.12–1	0.5	1				00.0	421.1	126.3	763.2	7100.0			
*Paecilomyces variotii* (10)	Manogepix	≤0.008	≤0.008	≤0.008	10100.0										
	Anidulafungin	≤0.008	≤0.008	≤0.008	10100.0										
	Micafungin	≤0.008	≤0.008	≤0.008	10100.0										
	Itraconazole	0.25–0.5	0.25	0.5					00.0	660.0	4100.0				
	Voriconazole	2–8	4	8								00.0	330.0	580.0	2100.0
	Amphotericin B	0.12–0.5	0.25	0.5				00.0	440.0	480.0	220.0				
*Purpureocillium lilacinus* (15) (formerly *Paecilomyces lilacinus*)	Manogepix	≤0.008–0.016	≤0.008	0.016	1173.3	4100.0									
	Anidulafungin	0.016–>4	0.12	0.5	00.0	16.7	113.3	440.0	253.3	480.0	293.3	093.3	093.3	093.3	1100.0
	Micafungin	0.016–>4	0.03	0.12	00.0	426.7	453.3	480.0	293.3	093.3	093.3	093.3	093.3	093.3	1100.0
	Itraconazole	0.5–>8	>8	>8						00.0	16.7	06.7	326.7	240.0	9100.0
	Voriconazole	0.06–1	0.25	0.5			00.0	16.7	06.7	966.7	493.3	1100.0			
	Amphotericin B	2–>2	>2	>2								00.0	16.7	06.7	14100.0
*Rasamsonia argillacea* species complex (12)^*k*^	Manogepix	≤0.008–0.016	≤0.008	0.016	866.7	4100.0									
	Anidulafungin	≤0.008–0.03	≤0.008	0.016	650.0	591.7	1100.0								
	Micafungin	≤0.008–0.016	≤0.008	≤0.008	1191.7	1100.0									
	Itraconazole	0.5*–*>8	2	>8						00.0	216.7	233.3	250.0	050.0	6100.0
	Voriconazole	8–>8	>8	>8										00.0	12100.0
	Amphotericin B	0.25–4	1	2					00.0	18.3	116.7	666.7	391.7	1100.0	
*Scedosporium* spp. (74)^*l*^	Manogepix	0.004–0.5	0.03	0.06	810.8	1936.5	3077.0	1495.9	298.6	098.6	1100.0				
	Anidulafungin	0.5–>4	4	>4						00.0	11.4	58.1	2035.1	3379.1	15100.0
	Micafungin	0.12–>4	0.5	>4				00.0	79.6	1935.6	2265.8	369.9	069.9	171.2	21100.0
	Itraconazole	2–>8	>8	>8								00.0	1216.2	421.6	58100.0
	Voriconazole	0.25–8	1	2					00.0	56.8	2439.2	3789.2	697.3	198.6	1100.0
	Amphotericin B	0.25–>2	>2	>2					00.0	22.7	25.4	816.2	1029.2	029.2	52100.0
*S. apiospermum/S. boydii* (58)	Manogepix	0.004–0.5	0.03	0.06	712.1	1639.7	2379.3	994.8	298.3	098.3	1100.0				
	Anidulafungin	0.5–>4	4	4						00.0	11.7	48.6	1839.7	3193.1	4100.0
	Micafungin	0.12–>4	0.5	>4				00.0	712.3	1742.1	2178.9	384.2	084.2	186.0	8100.0
	Itraconazole	2–>8	>8	>8								00.0	1017.2	322.4	45100.0
	Voriconazole	0.25–8	1	2					00.0	23.4	1936.2	3087.9	596.6	198.3	1100.0
	Amphotericin B	0.25–>2	>2	>2					00.0	23.4	26.9	820.7	1037.9	037.9	36100.0
*S. aurantiacum* (11)	Manogepix	0.016–0.06	0.03	0.06	00.0	218.2	563.6	4100.0							
	Anidulafungin	4–>4	>4	>4									00.0	218.2	9100.0
	Micafungin	>4	>4	>4										00.0	11100.0
	Itraconazole	8–>8	>8	>8										00.0	11100.0
	Voriconazole	0.25–1	1	1					00.0	19.1	445.5	6100.0			
	Amphotericin B	4–>4	>4	>4									00.0	19.1	10100.0

^*a*^ Contains *Aspergillus clavatus* (2), *A. flavus* species complex (173), *A. fumisynnematus* (1), *A. hortai* (1), *A. lentulus* (7), *A. melleus* (1), *A. nidulans* species complex (42), *A. niger* species complex (102), *A. nominus* (1), *A. ochraceus* species complex (1), *A. parasiticus* (3), *A. sclerotiorum* (3), *A. sydowii* (4), *A. tamarii* (3), *A. terreus* species complex (71), *A. thermomutatus* (2), *A. tubigensis* (23), *A. udagawae* (2), *A. unguis* (3), *A. ustus* species complex (12), *A. versicolor* (7), and *A. welwitschiae* (1).

^*b*^ Contains *A. flavus* (7) and *A. flavus* species complex (166).

^*c*^ Contains *A. nidulans* (29) and *A. nidulans* species complex (13).

^*d*^ Contains *A. niger* (83) and *A. niger* species complex (100).

^*e*^ Contains *A. terreus* (40) and *A. terreus* species complex (31).

^*f*^ Contains *A. ustus* species complex (12).

^*g*^ Contains *Fusarium annulatum* (2), *F. dimerum* species complex (1), *F. falciforme* (1), *F. incarnatum-equiseti* species complex (4), *F. oxysporum* species complex (8), *F. petroliphilum* (1), *F. solani* (2), *F. solani* species complex (27), and *F. (Gibberella) fujikuroi* species complex (21).

^*h*^ Contains *Fusarium solani* (2) and *F. solani* species complex (27).

^*i*^ Contains *Lichtheimia corymbifera* (8), *L. ramose* (1), *Mucor circinelloides* (8), *M. circinelloides*/*M. ramosissimus* (2), *M. inducus* (1), *Rhizomucor pusillus* (6), *Rhizopus microsporus* group (24), *R. oryzae* (12), and *R. oryzae* species complex (7).

^*j*^ Contains *Mucor circinelloides* (8), *M. circinelloides*/*M. ramosissimus* (2), and *M. inducus* (1).

^*k*^ Contains *Rasamsonia argillacea* (6) and *R. argillacea* species complex (6).

^*l*^ Contains *Scedosporium apiospermum* (9), *S. apiospermum*/*S. boydii* (37), *S. aurantiacum* (11), *S. dehoogii* (3), and *S. minutisporum* (2).

^*m*^ –, no value.

Of the 1,937 fungal clinical isolates tested, 776 (40.1%) were *Candida* spp. (26 species); 308 (15.9%) were non-*Candida* yeasts (48 species), including 179 *Cryptococcus neoformans* (formerly *C. neoformans* var. *grubii*); 548 (28.3%) were *Aspergillus* spp. (24 species); and 304 (15.7%) were other molds (41 species) (Tables 1 to 4; Tables S1 and S2).

### *In vitro* activity of manogepix against infrequently encountered yeast isolates

Among the 27 species of *Candida* listed in Table 1, manogepix was the most active against *Candida duobushaemulonii*, *Candida* (*Cyberlindnera*) *fabianii*, *Candida haemulonii*, *Candida intermedia*, *C*. (*Nakaseomyces*) *nivariensis*, *Candida pararugosa*, *Candida pelliculosa*, *C. pseudohaemulonii*, *C. theae* , and *Candida utilis* (MIC_50_, range ≤0.002–0.004 mg/L) and least active against *Candida inconspicua* (*Pichia cactophila*; MIC_50_, 2 mg/L), *C*. (*Pichia*) *norvegensis* (MIC_50_, 0.5 mg/L), and *C. kefyr* (*Kluyveromyces marxianus*; MIC_90_, 0.5 mg/L). All 77 *C*. *auris* isolates were inhibited by ≤0.06 mg/L of manogepix, and 90.9% were encompassed by the WT-UL of ≤0.03 mg/L.

Among the 776 isolates of uncommon species of *Candida* tested in this survey, 88.3% were inhibited by ≤0.06 mg/L and 96.9% inhibited by ≤0.25 mg/L of manogepix (Tables 1 and 2). Application of the manogepix WT-UL MIC cutoff values determined previously (28) demonstrates that most isolates of *C. dubliniensis* (99.5% WT), *C. orthopsilosis* (100.0% WT), *C*. (*Clavispora*) *lusitaniae* (97.3% WT), and *C. kefyr* (100.0% WT) display a WT phenotype for manogepix. There were 24 isolates for which the manogepix MIC value was ≥0.5 mg/L: *C. kefyr* (10 isolates), *C. lusitaniae* (2 isolates), *Candida norvegensis* (6 isolates), and *C. inconspicua* (6 isolates) (Table 1).

When compared to the *Candida* species, the potency of manogepix was reduced against isolates of *Cryptococcus* spp. (Table 1). Whereas 100.0% of isolates of *Cryptococcus deneoformans* (formerly *C. neoformans* var. *neoformans*) were inhibited by ≤1 mg/L of manogepix, 92.1% of *C. neoformans* (formerly *C. neoformans* var. *grubii*) and 83.3% of *Cryptococcus gattii* species complex were inhibited at this concentration. A single *C. gatti* species complex isolate had a manogepix MIC value of 2 mg/L (Table 1).

The remaining infrequently encountered yeast isolates notably include the antifungal-resistant genera *Trichosporon* spp., *Saprochaete* spp., and *Rhodotorula* spp. (Table 1). Both *Saprochaete* spp. and *Rhodotorula* spp. show susceptibility to manogepix that is comparable to that of the *Candida* spp. (MIC_90_ values of 0.06 and 0.12 mg/L, respectively). Manogepix is not active against the majority of *Trichosporon* species (*n* = 26; MIC_50/90_, >2/>2 mg/L). However, a single *Trichosporon capitatum* isolate had a manogepix MIC of 0.03 mg/L (Table 1). Among the remaining isolates of rare yeasts, isolates of *Blastobotrys adeninivorans*, *Hyphopichia burtonii*, *Kodamaea ohmeri*, *Lodderomyces elongisporus*, *Ogataea siamensis*, *Pichia kluyveri,* and *Saccharomyces cerevisiae* are all inhibited by 0.001–0.06 mg/L of manogepix (Table 1).

### *In vitro* activity of manogepix and comparators against infrequently encountered yeast isolates

Among 776 isolates of infrequently encountered species of *Candida*, manogepix (MIC_50/90_, 0.008/0.12 mg/L) and voriconazole (MIC_50/90_, ≤0.008/0.25 mg/L) were the most potent, and fluconazole (MIC_50/90_, 0.5/64 mg/L) was the least potent of the agents tested (Table 2). Overall, resistance to fluconazole was 16.1% followed by 11.1% resistance to anidulafungin, 6.8% resistance to voriconazole, 2.2% resistance to amphotericin B, and 2.2% resistance to micafungin using current CLSI M27M44S (CLSI, 2022b) breakpoint interpretive criteria for *C. albicans* for comparative purposes (Table 2).

The 776 isolates of infrequently encountered *Candida* spp. included 24 isolates (3.1%) of 4 different species [*C. kefyr* (10 isolates), *C. lusitaniae* (2 isolates), *C. norvegensis* (6 isolates), and *C. inconspicua* (6 isolates)] for which the manogepix MIC values were elevated at 0.5 mg/L or greater (range, 0.5–2 mg/L) (Table 1). Two of these species, *C. norvegensis* and *C. inconspicua*, are closely related to *C. krusei*, a species known for its intrinsic resistance not only to fluconazole but also to manogepix. Whereas none of the isolates of *C. kefyr* were resistant to fluconazole using the CLSI *C. albicans* clinical breakpoints, two *C*. *lusitaniae*, six *C*. *norvegensis,* and six *C*. *inconspicua* isolates for which the manogepix MIC values were ≥0.5 mg/L were also resistant to fluconazole (MIC range, 8–32 mg/L).

All (100.0%) of the 77 *C*. *auris* isolates were inhibited by manogepix at ≤0.06 mg/L, and 90.9% were inhibited at the WT-UL MIC cutoff value of ≤0.03 mg/L (Tables 1 and 2). Using the CDC tentative clinical breakpoints, 85.7% of the isolates were resistant to fluconazole, 14.3% were resistant to amphotericin B, and 1.3% were resistant to anidulafungin and micafungin (Table 2). Application of the CLSI-resistant breakpoints for *C. albicans* to this collection showed 89.6% were resistant to fluconazole, 51.9% were resistant to voriconazole, 14.3% were resistant to amphotericin B, 5.2% were resistant to anidulafungin, and 1.3% were resistant to micafungin. Notably, 58.0% of fluconazole-resistant isolates were also resistant to voriconazole. Of the 77 isolates of *C. auris*, 22 (28.6%) were from North America (5 states), 32 (41.6%) were from Europe (4 countries, 5 sites), 22 (28.6%) were from Latin American [1 country (Panama), 1 site], and 1 (1.3%) was from the Asia-Pacific region [1 country (Japan), 1 site]. The antifungal resistance/non-wild type (NWT) varied considerably by geographic region. Whereas none of the isolates from Latin America (Panama) or the single isolate from Japan expressed an NWT manogepix phenotype [MIC >0.03 mg/L (> WT Ul)], 6.2% of the isolates from Europe and 22.7% of the isolates from North America were NWT to manogepix. Resistance to fluconazole and amphotericin B was detected in isolates from North America (90.9% fluconazole resistant and 36.4% amphotericin B resistant), Europe (100.0% fluconazole resistant and 6.2% amphotericin B resistant), and Latin America (59.1% fluconazole resistant and 4.5% amphotericin B resistant) (data not shown). Aside from the Latin American isolates (4.5% resistant to anidulafungin and micafungin), none of the remaining isolates of *C. auris* in this survey were resistant to the echinocandins. The single *C. auris* isolate from the Asia-Pacific region was WT to manogepix, resistant to fluconazole, and susceptible to the echinocandins and amphotericin B.

Manogepix inhibited 99.1% of the 221 isolates of *C. dubliniensis* at the WT-UL of 0.008 mg/L (Tables 1 and 2). Rates of resistance to the comparator antifungal agents were low when applying the CLSI *C. albicans* interpretive criteria: fluconazole (0.5% resistant), voriconazole (0.0% resistant), anidulafungin (0.0% resistant), micafungin (0.9% resistant), and amphotericin B (0.0% resistant) (Table 2).

Aside from *C. fabianii* and *C. kefyr*, the remaining species of rare *Candida* shown in Table 2 exhibited decreased susceptibility to fluconazole. Neither *C. fabianii* nor *C. kefyr* showed resistance to fluconazole, voriconazole, or the echinocandins, whereas reduced susceptibility to fluconazole was detected in isolates of *Candida fermentati* (*Meyerozyma caribbica*; 30.3% resistant), *C*. (*Meyerozyma*) *guilliermondii* (29.6% resistant), *C. lusitaniae* (6.7% resistant), *Candida metapsilosis* (5.9% resistant), *C. orthopsilosis* (9.1% resistant), and *C. pelliculosa* (resistant) (Table 2). Among these isolates, *C. fermentati* (9.1% resistant), *C. guilliermondii* (22.2% resistant), *C. orthopsilosis* (3.0% resistant), and *C. pelliculosa* (7.7% resistant) also showed decreased susceptibility to voriconazole, and *C. fermentati* [60.6% resistant (anidulafungin)], *C. guilliermondii* [100.0% resistant (anidulafungin); 18.5% resistant (micafungin)], *C. lusitaniae* [4.0% resistant (anidulafungin)], *C. metapsilosis* [2.9% resistant (micafungin)], and *C. orthopsilosis* [36.4% resistant (anidulafungin); 7.6% resistant (micafungin)] expressed potential resistance to echinocandins (Table 2). Aside from *C. auris*, only *C. kefyr* (1.3% resistant) showed resistance to amphotericin B.

The isolates of *Cryptococcus* spp. (*n* = 199) in this survey include 6 of *C. gattii* species complex, 1 of *Cryptococcus* (*Papiliotrema*) *laurentii*, 178 of *C. neoformans,* and 13 of *C. deneoformans* (Tables 1 and 2). The cryptococci are intrinsically resistant to the echinocandins and are generally susceptible to the azoles and amphotericin B. Overall, 98.5% of the isolates tested were inhibited by ≤2 mg/L of manogepix. The three isolates for which the manogepix MIC value was 4 mg/L were all *C. neoformans* and were WT to fluconazole. The two most common species groups of *Cryptococcus* in this survey were *C. neoformans* (*n* = 178) and *C. deneoformans* (*n* = 13). Both species showed a predominantly WT profile to fluconazole [98.9% and 100.0% WT (MIC ≤8 mg/L), respectively], voriconazole [100.0% WT (MIC ≤0.25 mg/L) for both], and amphotericin B (all isolates MIC ≤1 mg/L). The six isolates of *C. gattii* species complex were all inhibited by ≤2 mg/L of manogepix and were WT to fluconazole, voriconazole, and amphotericin B (Table S1).

Some of the more notable and potentially MDR genera of non-*Candida* yeasts are the *Saprochaete* (*Magnusiomyces*) spp. (*Saprochaete clavata* and *S. capitata*), *Rhodotorula mucilaginosa,* and *Saccharomyces cerevisiae* (Tables 1, 2, and S1). All of these species are inhibited by low concentrations of manogepix, are susceptible/WT to amphotericin B, and show decreased susceptibility to fluconazole [percent of isolates with fluconazole MIC values ≥8 mg/L: *Saprochaete* spp. (81.2%), *R. mucilaginosa* (94.4%), and *S. cerevisiae* (25.0%)]. Both *Saprochaete* and *Rhodotorula* spp. are intrinsically resistant to the echinocandins.

The *Trichosporon* spp., dominated by *T. asahii,* are well known to exhibit intrinsic resistance to the echinocandins and reduced susceptibility (MIC ≥8 mg/L) to fluconazole (Tables 1, 2, and S1 ). *T. asahii* shows decreased susceptibility to manogepix (53.8% MIC >2 mg/L) and resistance to fluconazole (34.6% MIC ≥8 mg/L) and voriconazole (11.5% MIC ≥1 mg/L). *T*. (*Cutaneotrichosporon*) *mucoides* and *T*. (*Apiotrichum*) *mycotoxinivorans*, and *T. inkin* isolates show a similar profile to that of *T. asahii*. A single *Trichosporon capitatum* isolate exhibited a manogepix MIC of 0.03 mg/L.

### *In vitro* activity of manogepix against infrequently encountered mold isolates

The rare molds included in this survey were 24 species or species complexes of *Aspergillus* [non-*A*. *fumigatus* (*n* = 548 isolates)] and 41 species/species complexes of non-*Aspergillus* molds (*n* = 304 isolates) (Tables 3 and 4).

The 548 isolates of *Aspergillus* originated from Europe [201 isolates (36.7%)], North America [201 isolates (36.7%)], the Asia-Pacific region [136 isolates (24.8%)], and Latin America [10 isolates (1.8%)] (data not shown). Among the isolates of *Aspergillus*, 543 (99.1%) were inhibited by ≤0.06 mg/L of manogepix (Table 4). All (100.0%) of the *Aspergillus* isolates from APAC and LA were inhibited by ≤0.06 mg/L of manogepix, compared to 99.0% and 98.5% of isolates from NA and EU, respectively. There were only five isolates for which the manogepix MIC values were greater than 0.06 mg/L. These isolates represented five different locations: Italy (*Aspergillus thermomutatus*), Turkey (*A. flavus* species complex), France (*Aspergillus ochraceus* species complex), Kansas, USA (*A. flavus* species complex), and Massachusetts, USA (*A. niger* species complex).

The most common non-*fumigatus* species of *Aspergillus* in this 2017–2022 survey that were tested against manogepix included the following four *Aspergillus* species complex in the order of frequency: *A. niger* species complex (*n* = 185), *A. flavus* species complex (*n* = 173), *A. terreus* species complex (*n* = 71), and *Aspergillus nidulans* species complex (*n* = 42) (Tables 3 and 4). Manogepix exhibited potent *in vitro* activity against all four *Aspergillus* species complex shown in Tables 3 and 4, with MEC_90_ values of 0.016–0.06 mg/L. The WT-UL determined previously for three of these groups was ≤0.03 mg/L for *A. niger* species complex (95.7% WT) and *A. terreus* species complex (100.0% WT) and ≤0.06 mg/L for *A. flavus* species complex (98.8% WT) (Tables 3 and 4).

Manogepix was notably active against many less common species of *Aspergillus,* including *Aspergillus tubingensis* (MEC_90_, 0.03 mg/L), *A. ustus* species complex (MEC_90_, 0.016 mg/L), *A. lentulus* (MEC range, 0.008–0.016 mg/L), *A. sclerotiorum* (MEC range, 0.016–0.03 mg/L), *A. thermomutatus* (MEC range, 0.06–0.25 mg/L), *A. udagawae* (MEC, 0.016 mg/L), *A. versicolor* (MEC range, ≤0.002–0.03 mg/L), among others (Tables 3 and 4).

The activity of manogepix against the often antifungal-resistant species *Exophiala dermatitidis*, *Fusarium* spp., *Lomentospora prolificans*, *Paecilomyces variotii*, *Purpureocillium lilacinum*, *Rasamsonia argillacea* species complex, and *Scedosporium* spp. was comparable to that of the rare species of *Aspergillus* with MEC_50/90_ values ranging from ≤0.008 to 0.03/≤0.008 to 0.06 mg/L (Tables 3 and 4). In contrast, the Mucorales were considerably less susceptible to manogepix with overall MEC_90_ values >4 mg/L (52.2%), with little variation across the different genera (Tables 4 and 2). Among the remaining 25 miscellaneous molds (Table S2), most isolates (84.0%) were inhibited by ≤0.12 mg/L of manogepix. Manogepix was least active (MEC ≥0.25 mg/L) against *Alternaria alternata, Pseudopithomyces sacchari,* and *Verruconis gallopava* (Tables 3; Table S2).

### *In vitro* activity of manogepix and comparators against infrequently encountered mold isolates

Manogepix, anidulafungin, and micafungin showed similar activities against 548 non-*A*. *fumigatus* isolates of *Aspergillus* (MEC_90_, 0.03 mg/L for all three agents) (Table 4). Both amphotericin B and voriconazole inhibited 96.0% of isolates at an MIC value of 2 mg/L or less (Table 4), whereas itraconazole was slightly less active (85.9% at MEC ≤2 mg/L).

Among the six species/species complexes of *Aspergillus* listed in Tables 3 and 4. *A*. *flavus* species complex, *A. nidulans* species complex, *A. niger* species complex, and *A. terreus* species complex all expressed a predominantly (>95%) WT phenotype for manogepix, the echinocandins, itraconazole, voriconazole, and amphotericin B. In contrast, both *A. ustus* species complex and *A. tubingensis* show decreased susceptibility to itraconazole (MEC_90_, >8 mg/L for both species complexes) and voriconazole (MEC_90_ ≥8 and 4 mg/L, respectively) (Tables 3 and 4). All isolates of *A. tubingensis* and 83.3% of *A. ustus* species complex were inhibited by ≤2 mg/L of amphotericin B.

The non-*Aspergillus* molds include a diverse array of different species, most of which show decreased susceptibility to one or more classes of systemically active antifungal agents (Tables 4 and S2). The Mucorales as a whole show decreased susceptibility to manogepix (MEC_90_, >4 mg/L), the echinocandins (MEC_90_, >4 mg/L), itraconazole (MIC_90_, 8 mg/L), and voriconazole (MEC_90_, >8 mg/L). Amphotericin B is reliably active against these organisms (MIC_90_, 1 mg/L) (Table 4). The *Fusarium* spp. and *Lomentospora prolificans* demonstrate striking resistance profiles with decreased susceptibility to echinocandins, azoles, and amphotericin B (Table 4). All isolates of *Fusarium solani* species complex (MEC_90_, 0.03 mg/L) and *F*. (*Gibberella*) *fujikuroi* (MEC_90_, 0.03 mg/L) were inhibited by ≤0.12 mg/L of manogepix, like that of the *Aspergillus* spp. (Tables 3 and 4). *L. prolificans* isolates were all resistant to azoles (MIC_90_, >8 mg/L), echinocandins (MIC_90_, >4 mg/L), and amphotericin B (MIC_90_, ≥2 mg/L) yet showed low MEC values to manogepix (MIC_90_, 0.06 mg/L). Isolates of *P. variotii*, *Purpureocillium lilacinum,* and *Rasamsonia argillacea* species complex generally show low MEC values for manogepix and the echinocandins but are variable in their susceptibility to itraconazole, voriconazole, and amphotericin B (Table 4). *R. argillacea* species complex exhibits decreased susceptibility to both itraconazole and voriconazole (MIC_90_ values, >8 mg/L), *P. variotii* shows decreased susceptibility to voriconazole (MIC_90_, 8 mg/L) but not itraconazole (MIC_90_, 0.5 mg/L), and *P. lilacinum* is resistant to itraconazole (MIC_90_, >8 mg/L) but not voriconazole (MIC_90_, 0.5 mg/L) (Table 4). *P. lilacinus* is also intrinsically resistant to amphotericin B (MIC_90_, >2 mg/L). The *Scedosporium* spp. show broad resistance to echinocandins (MEC_90_, >4 mg/L), itraconazole (MIC_90_, >8 mg/L), and amphotericin B (MIC_90_, >2 mg/L) but are considered to be susceptible to voriconazole (MIC_90_, 2 mg/L); the manogepix MEC values are low (MEC_90_, 0.06 mg/L).

The susceptibility data for manogepix and comparators against non-*Aspergillus* molds with less than 10 isolates each are shown in Table S2. These isolates encompass 19 different species/species complexes and show decreased susceptibility to echinocandins (*C. cinerea, Exophiala attenuata, Medicopsis romeroi, M. cirrosus, Pleurostomophora richardsiae, S. kiliense,* and *Scopulariopsis* spp.), voriconazole (*Medicopsis romeroi, M. cirrosus, Monascus ruber, Penicillium citrinum, S. kiliense*, and *Scopulariopsis* spp.), and amphotericin B (*Exophiala attenuata, M. cirrosus, S. kiliense,* and *Scopulariopsis* spp.). Notably, all these extremely uncommon fungi except *A. alternata* (MEC, 1 mg/L), *Pseudopithomyces sacchari* (MEC, 0.25 mg/L), and *V. gallopava* (MEC, 0.5–1 mg/L) exhibit low MEC values (≤0.12 mg/L) to manogepix (Table S2).

## DISCUSSION

In recent years, the incidence of unusual yeasts and molds in the immunosuppressed patient population has increased, suggesting a shift in the epidemiology of IFIs (1–5, 12, 48). The opportunistic fungal pathogen spectrum is increasing as the population of individuals at risk for IFI expands beyond hematopoietic stem cell transplantation (HSCT) and solid organ transplantation to include those receiving monoclonal antibody treatment for immunosuppression as well as those individuals suffering from severe respiratory viral infections (e.g., influenza and/or SARS-CoV-2) and the elderly (2–5, 48–50). The application of molecular and proteomic methods for fungal identification and resistance testing has allowed clinical microbiology laboratories to characterize even rare fungi to a degree not possible 20 years ago (21).

Current antifungal agents cover most opportunistic fungal pathogens, including but not limited to *C. albicans*, *A. fumigatus*, and *C. neoformans* (51); however, breakthrough infections do happen and increasingly include relatively uncommon yeasts and molds (8, 15). The use of mold-active antifungal agents for fungal prophylaxis in individuals at high risk for IFI has reduced the frequency of candidemia and invasive aspergillosis (IA) (4, 8, 15); however, this selective pressure has resulted in an increase in breakthrough infections caused by infrequently encountered species of *Candida* (*C. auris, C. guilliermondii, and C. rugosa*), and *Aspergillus* (*A. lentulus, A. udagawae,* and *A. ustus* species complex), non-*Candida* yeasts (*Saprochaete, Rhodotorula, Trichosporon*, and *Saccharomyces* spp.), and non-*Aspergillus* molds (Mucorales, *L. prolificans, Fusarium,* and *Scedosporium* spp.) (18). Optimal treatment regimens against unusual fungal pathogens have not been established, and anecdotal reports of successes have been published with various agents alone or in combination (8, 15). Some of these fungi (e.g., *Fusarium, Scedosporium, Lomentospora, Trichosporon, and C. auris*) are among the most drug-resistant fungal organisms encountered in clinical practice (8, 15). It is worth noting that, in very high-risk patients (e.g., HSCT), the organisms with the greatest intrinsic resistance to the antifungal agent administered will ultimately emerge as a cause of infection (52).

The results of the present survey confirm and extend those previously reported in 2017 (28, 36, 37), regarding the *in vitro* activities of manogepix and comparator antifungals against contemporary yeast and mold pathogens. In this survey, we have focused on the less frequently encountered yeasts and molds, demonstrating decreased susceptibility of the rare yeasts to azoles and echinocandins, and of the rare molds to the azoles. Resistance to amphotericin B is difficult to characterize but appears to be intrinsic to several species such as *A. lentulus, A. terreus* species complex, *Scedosporium* spp., *L. prolificans,* among others (Tables 2 and 4).

The resistance profiles shown in Table 2 for the rare yeasts document frequent resistance to fluconazole among the *Candida* and non-*Candida* yeast species and intrinsic resistance to the echinocandins among *Trichosporon, Saprochaete,* and *Rhodotorula* spp. (Table 2). Voriconazole is active against most species of *Candida* (MIC_90_ 0.25 mg/L) except *C. auris* (MIC_90_, 2 mg/L), and *C. guilliermondii* (MIC_90_, 4 mg/L) and against the non-*Candida* yeasts (MIC_90_, range 0.06–0.25 mg/L) except for *R. mucilaginosa* (MIC_90_, 2 mg/L) and *Trichosporon* spp. (MIC_90_, 1 mg/L). Elevated MIC values for the echinocandins (MIC >1 mg/L) were seen with *C. fermentati* [MIC_90_, 2 mg/L (anidulafungin)], *C. guilliermondii* [MIC_90_, 2 mg/L (anidulafungin)], and *C. orthopsilosis* [MIC_90_, 2 mg/L (anidulafungin)]. The echinocandins show good activity against *S. cerevisiae* [MIC_90_, 0.5 mg/L (anidulafungin)] but are inactive against the other rare yeasts shown in Table 2.

Whereas most of the non-*A*. *fumigatus* species of *Aspergillus* expressed a WT phenotype for the echinocandins, azoles, and amphotericin B, both *A. ustus* species complex and *A. tubingensis* show decreased susceptibility to itraconazole (MEC_90_, >8 mg/L for both) and voriconazole (MEC_90_, 8 and 4 mg/L, respectively) (Table 4). The non-*Aspergillus* molds generally show resistance or decreased susceptibility to one or more classes of antifungal agents (Table 4; Table S2). The Mucorales show resistance to echinocandins, itraconazole, and voriconazole but not amphotericin B. *Fusarium* spp. and *L. prolificans* are intrinsically resistant to echinocandins, azoles, and amphotericin B, although voriconazole with or without a second agent in combination is often used in treatment of infections due to these organisms (15). *Scedosporium* spp. are resistant to amphotericin B, and isolates of *P. variotii*, *Purpureocillium lilacinum,* and *Rasamsonia argillacea* species complex generally show low MEC values for the echinocandins but are variable in their susceptibility to itraconazole, voriconazole, and amphotericin B (Table 4).

The *in vitro* susceptibility of the infrequently encountered yeasts and molds to manogepix in the present survey expands upon the data presented previously and documents the WT manogepix MIC distribution for several uncommon, and drug-resistant, fungi (28, 36, 37). In previous surveys of the *in vitro* activity of manogepix against yeasts and molds, small numbers of the less common species were included but not displayed in detail. We have expanded our analysis in the present survey to include 1,937 isolates of the less commonly encountered fungi and confirm the excellent potency and spectrum of manogepix against azole- and echinocandin-resistant yeasts and azole-resistant molds. Notably, we show that manogepix is active (MIC/MEC, ≤0.12 mg/L) against several MDR fungi, including *C. auris*, *Saprochaete* spp., *Rhodotorula* spp., *A. terreus* species complex, *A. ustus* species complex, *Fusarium* spp., *Scedosporium* spp., and *L. prolificans. C. krusei* is known to be intrinsically resistant to manogepix, and it appears from this survey that the closely related species *C. inconspicua* and *C. norvegensis* also may be as well. The only other rare yeast that may not be a target for manogepix is *Trichosporon* spp. (MIC_50/90_, >2/>2 mg/L). Manogepix shows potent activity against the uncommon species of *Aspergillus*, including antifungal-resistant species such as *A. lentulus* and *A. ustus* species complex. The Mucorales (MEC_90_, >4 mg/L) are considerably less susceptible to manogepix when compared to the rare species of *Aspergillus* (MEC_90_, 0.03 mg/L) (Table 4).

The broad spectrum of manogepix is notable for its activity against many less common and often antifungal-resistant yeast and mold strains. Clinical development of the prodrug fosmanogepix has focused on the treatment of infections due to *Candida*, *Aspergillus*, and the rare molds. Phase 1 studies in healthy volunteers have demonstrated high (>90%) oral bioavailability of manogepix and maintenance of manogepix plasma drug exposures above estimated antifungal target levels for 7–42 days, even when switching from intravenous (IV) to oral dosing (32). Following IV administration of 600 mg fosmanogepix over a 3-hour infusion period, the observed geometric manogepix *C*_max_ on day 7 was 7.9 mg/L and on day 14 was 6.3 mg/L (53). These exposures were similar with either IV or oral administration in both healthy volunteers and patients with neutropenia (53). The US Food and Drug Administration has granted Fast Track designation for intravenous and oral formulations of fosmanogepix for seven different indications, including treatment of invasive candidiasis, invasive aspergillosis, scedosporiosis, fusariosis, mucormycosis, cryptococcosis, and coccidioidomycosis (32). Three phase 2 clinical trials have been completed. The first (NCT03604705; clinicaltrials.gov) was an open-label, non-comparative study assessing the safety and efficacy of fosmanogepix in the treatment of non-neutropenic patients with candidemia which met its primary efficacy endpoint with a treatment success rate of 80% with an acceptable safety profile (54). The second study (NCT04148287; clinicaltrials.gov) tested the safety and efficacy of fosmanogepix treatment in patients with candidemia caused by *C. auris* (55). Treatment success at the end of study treatment and day 30 survival were 89% with no treatment-related adverse events or study drug discontinuations reported. The third study (NCT04240886) examined the use of fosmanogepix for the treatment of patients with invasive mold infections caused by *Aspergillus* species or rare molds. Results of this study are awaiting publication. Continued development of fosmanogepix for the treatment of IFI, including MDR strains, is warranted.

## SUPPLEMENTAL MATERIAL

Table S1 aac.01132-23-s0001.docx Antimicrobial activity of manogepix and comparators against infrequently encountered yeast isolates.

Table S2 aac.01132-23-s0002.docx Antimicrobial activity of manogepix and comparators against infrequently encountered mold isolates.

ASM does not own the copyrights to Supplemental Material that may be linked to, or accessed through, an article. The authors have granted ASM a non-exclusive, world-wide license to publish the Supplemental Material files. Please contact the corresponding author directly for reuse.
