# Supplementary material for: In vitro activity of manogepix and comparators against infrequently encountered yeast and mold isolates from the SENTRY Surveillance Program (2017–2022)
Source: Antimicrob Agents Chemother. 2024 Jan 11;68(2):e01132-23. doi: 10.1128/aac.01132-23 (PMC10848754; doi:10.1128/aac.01132-23)
Supplement: Table S1 — Antimicrobial activity of manogepix and comparators against infrequently encountered yeast isolates. [file aac.01132-23-s0001.docx]

Table S1 Antimicrobial activity of manogepix and comparator agents against infrequently encountered yeast isolates

| **Organism/organism group (no. of isolates)** | **No. and cumulative % of isolates inhibited at MIC (mg/L) of:** | | | | | | | | | | | | | | | | | | | |  | **MIC_50_** | **MIC_90_** |
| --- | --- | --- | --- | --- | --- | --- | --- | --- | --- | --- | --- | --- | --- | --- | --- | --- | --- | --- | --- | --- | --- | --- | --- |
|  | **≤0.0005** | **0.001** | **0.002** | **0.004** | **0.008** | **0.015** | **0.03** | **0.06** | **0.12** | **0.25** | **0.5** | **1** | **2** | **4** | **8** | **16** | **32** | **64** | **128** | **> ^a^** |  |  |  |
| *Apiotrichum mycotoxinivorans*  (*Trichosporon mycotoxinivorans*) | | | | | | | | | | | | | | | | | | | | | | |  |
| Manogepix (5) |  |  |  |  |  |  |  |  |  | 0 0.0 | 1 20.0 | 0 20.0 | 0 20.0 |  |  |  |  |  |  | 4 100.0 |  | >2 |  |
| Fluconazole (5) |  |  |  |  |  |  |  |  |  |  | 0 0.0 | 1 20.0 | 0 20.0 | 2 60.0 | 1 80.0 | 1 100.0 |  |  |  |  |  | 4 |  |
| Voriconazole (5) |  |  |  |  | 0 0.0 | 1 20.0 | 0 20.0 | 1 40.0 | 0 40.0 | 2 80.0 | 0 80.0 | 1 100.0 |  |  |  |  |  |  |  |  |  | 0.25 |  |
| Anidulafungin (5) |  |  |  |  |  |  |  |  |  |  |  |  |  | 0 0.0 |  |  |  |  |  | 5 100.0 |  | >4 |  |
| Micafungin (5) |  |  |  |  |  |  |  |  |  |  |  |  |  | 0 0.0 |  |  |  |  |  | 5 100.0 |  | >4 |  |
| Amphotericin B (5) |  |  |  |  |  |  |  |  |  |  | 0 0.0 | 2 40.0 | 3 100.0 |  |  |  |  |  |  |  |  | 2 |  |
|  | | | | | | | | | | | | | | | | | | | | | | | |
| *Blastobotrys adeninivorans* | | | | | | | | | | | | | | | | | | | | | | |  |
| Manogepix (1) |  |  | 0 0.0 | 1 100.0 |  |  |  |  |  |  |  |  |  |  |  |  |  |  |  |  |  |  |  |
| Fluconazole (1) |  |  |  |  |  |  |  |  |  |  |  |  |  |  |  |  | 0 0.0 | 1 100.0 |  |  |  |  |  |
| Voriconazole (1) |  |  |  |  |  |  |  |  |  |  | 0 0.0 | 1 100.0 |  |  |  |  |  |  |  |  |  |  |  |
| Anidulafungin (1) |  |  |  |  |  |  |  |  | 0 0.0 | 1 100.0 |  |  |  |  |  |  |  |  |  |  |  |  |  |
| Micafungin (1) |  |  |  |  |  |  | 0 0.0 | 1 100.0 |  |  |  |  |  |  |  |  |  |  |  |  |  |  |  |
| Amphotericin B (1) |  |  |  |  |  |  |  |  |  |  | 0 0.0 | 1 100.0 |  |  |  |  |  |  |  |  |  |  |  |
|  | | | | | | | | | | | | | | | | | | | | | | | |
| *Candida bracarensis*  (*Nakaseomyces bracarensis*) | | | | | | | | | | | | | | | | | | | | | | |  |
| Manogepix (4) |  |  | 0 0.0 | 1 25.0 | 1 50.0 | 1 75.0 | 1 100.0 |  |  |  |  |  |  |  |  |  |  |  |  |  |  | 0.008 |  |
| Fluconazole (4) |  |  |  |  |  |  |  |  |  | 0 0.0 | 1 25.0 | 1 50.0 | 1 75.0 | 1 100.0 |  |  |  |  |  |  |  | 1 |  |
| Voriconazole (4) |  |  | 0 0.0 | 1 25.0 | 0 25.0 | 0 25.0 | 2 75.0 | 1 100.0 |  |  |  |  |  |  |  |  |  |  |  |  |  | 0.03 |  |
| Anidulafungin (4) |  |  |  |  |  |  | 0 0.0 | 1 25.0 | 2 75.0 | 0 75.0 | 0 75.0 | 0 75.0 | 1 100.0 |  |  |  |  |  |  |  |  | 0.12 |  |
| Micafungin (4) |  |  |  |  | 0 0.0 | 2 50.0 | 1 75.0 | 0 75.0 | 0 75.0 | 0 75.0 | 1 100.0 |  |  |  |  |  |  |  |  |  |  | 0.015 |  |
| Amphotericin B (4) |  |  |  |  |  |  |  |  |  | 0 0.0 | 3 75.0 | 1 100.0 |  |  |  |  |  |  |  |  |  | 0.5 |  |
|  | | | | | | | | | | | | | | | | | | | | | | | |
| *Candida duobushaemulonii* | | | | | | | | | | | | | | | | | | | | | | |  |
| Manogepix (4) |  |  | 2 50.0 | 2 100.0 |  |  |  |  |  |  |  |  |  |  |  |  |  |  |  |  |  | ≤0.002 |  |
| Fluconazole (4) |  |  |  |  |  |  |  |  |  |  |  | 0 0.0 | 1 25.0 | 1 50.0 | 0 50.0 | 0 50.0 | 0 50.0 | 1 75.0 | 1 100.0 |  |  | 4 |  |
| Voriconazole (4) |  |  |  |  |  | 0 0.0 | 2 50.0 | 0 50.0 | 0 50.0 | 0 50.0 | 2 100.0 |  |  |  |  |  |  |  |  |  |  | 0.03 |  |
| Anidulafungin (4) |  |  |  |  |  |  |  | 0 0.0 | 2 50.0 | 1 75.0 | 1 100.0 |  |  |  |  |  |  |  |  |  |  | 0.12 |  |
| Micafungin (4) |  |  |  |  |  |  | 0 0.0 | 2 50.0 | 2 100.0 |  |  |  |  |  |  |  |  |  |  |  |  | 0.06 |  |
| Amphotericin B (4) |  |  |  |  |  |  |  |  |  |  |  | 0 0.0 | 4 100.0 |  |  |  |  |  |  |  |  | 2 |  |
|  | | | | | | | | | | | | | | | | | | | | | | | |
| *Candida haemulonii* | | | | | | | | | | | | | | | | | | | | | | |  |
| Manogepix (6) |  |  | 5 83.3 | 1 100.0 |  |  |  |  |  |  |  |  |  |  |  |  |  |  |  |  |  | ≤0.002 |  |
| Fluconazole (6) |  |  |  |  |  |  |  |  |  | 0 0.0 | 1 16.7 | 0 16.7 | 1 33.3 | 3 83.3 | 1 100.0 |  |  |  |  |  |  | 4 |  |
| Voriconazole (6) |  |  |  | 0 0.0 | 1 16.7 | 0 16.7 | 1 33.3 | 1 50.0 | 3 100.0 |  |  |  |  |  |  |  |  |  |  |  |  | 0.06 |  |
| Anidulafungin (6) |  |  |  |  |  |  | 0 0.0 | 1 16.7 | 1 33.3 | 3 83.3 | 1 100.0 |  |  |  |  |  |  |  |  |  |  | 0.25 |  |
| Micafungin (6) |  |  |  |  |  |  | 0 0.0 | 2 33.3 | 3 83.3 | 1 100.0 |  |  |  |  |  |  |  |  |  |  |  | 0.12 |  |
| Amphotericin B (6) |  |  |  |  |  |  |  |  |  | 0 0.0 | 1 16.7 | 5 100.0 |  |  |  |  |  |  |  |  |  | 1 |  |
|  | | | | | | | | | | | | | | | | | | | | | | | |
| *Candida inconspicua*  (*Pichia cactophila*) | | | | | | | | | | | | | | | | | | | | | | |  |
| Manogepix (6) |  |  |  |  |  |  |  |  |  | 0 0.0 | 1 16.7 | 0 16.7 | 5 100.0 |  |  |  |  |  |  |  |  | 2 |  |
| Fluconazole (6) |  |  |  |  |  |  |  |  |  |  |  |  |  |  | 0 0.0 | 3 50.0 | 3 100.0 |  |  |  |  | 16 |  |
| Voriconazole (6) |  |  |  |  |  |  |  | 0 0.0 | 5 83.3 | 1 100.0 |  |  |  |  |  |  |  |  |  |  |  | 0.12 |  |
| Anidulafungin (6) |  |  |  | 0 0.0 | 2 33.3 | 1 50.0 | 2 83.3 | 1 100.0 |  |  |  |  |  |  |  |  |  |  |  |  |  | 0.015 |  |
| Micafungin (6) |  |  |  |  | 0 0.0 | 5 83.3 | 1 100.0 |  |  |  |  |  |  |  |  |  |  |  |  |  |  | 0.015 |  |
| Amphotericin B (6) |  |  |  |  |  |  |  |  | 0 0.0 | 3 50.0 | 3 100.0 |  |  |  |  |  |  |  |  |  |  | 0.25 |  |
|  | | | | | | | | | | | | | | | | | | | | | | | |
| *Candida intermedia* | | | | | | | | | | | | | | | | | | | | | | |  |
| Manogepix (2) |  |  | 0 0.0 | 1 50.0 | 0 50.0 | 0 50.0 | 1 100.0 |  |  |  |  |  |  |  |  |  |  |  |  |  |  | 0.004 |  |
| Fluconazole (2) |  |  |  |  |  |  |  |  |  | 0 0.0 | 1 50.0 | 1 100.0 |  |  |  |  |  |  |  |  |  | 0.5 |  |
| Voriconazole (2) |  |  |  |  | 0 0.0 | 2 100.0 |  |  |  |  |  |  |  |  |  |  |  |  |  |  |  | 0.015 |  |
| Anidulafungin (2) |  |  |  |  | 0 0.0 | 1 50.0 | 0 50.0 | 0 50.0 | 1 100.0 |  |  |  |  |  |  |  |  |  |  |  |  | 0.015 |  |
| Micafungin (2) |  |  |  |  |  | 0 0.0 | 1 50.0 | 1 100.0 |  |  |  |  |  |  |  |  |  |  |  |  |  | 0.03 |  |
| Amphotericin B (2) |  |  |  |  |  |  |  |  | 0 0.0 | 2 100.0 |  |  |  |  |  |  |  |  |  |  |  | 0.25 |  |
|  | | | | | | | | | | | | | | | | | | | | | | | |
| *Candida lipolytica*  (*Yarrowia lipolytica*) | | | | | | | | | | | | | | | | | | | | | | |  |
| Manogepix (7) |  |  |  | 0 0.0 | 1 14.3 | 2 42.9 | 3 85.7 | 1 100.0 |  |  |  |  |  |  |  |  |  |  |  |  |  | 0.03 |  |
| Fluconazole (7) |  |  |  |  |  |  |  |  |  | 0 0.0 | 2 28.6 | 2 57.1 | 2 85.7 | 1 100.0 |  |  |  |  |  |  |  | 1 |  |
| Voriconazole (7) |  |  |  |  | 0 0.0 | 3 42.9 | 3 85.7 | 1 100.0 |  |  |  |  |  |  |  |  |  |  |  |  |  | 0.03 |  |
| Anidulafungin (7) |  |  |  |  |  | 0 0.0 | 1 14.3 | 0 14.3 | 0 14.3 | 3 57.1 | 2 85.7 | 1 100.0 |  |  |  |  |  |  |  |  |  | 0.25 |  |
| Micafungin (7) |  |  |  |  |  |  |  |  | 0 0.0 | 1 14.3 | 5 85.7 | 1 100.0 |  |  |  |  |  |  |  |  |  | 0.5 |  |
| Amphotericin B (7) |  |  |  |  |  |  |  |  |  | 0 0.0 | 2 28.6 | 5 100.0 |  |  |  |  |  |  |  |  |  | 1 |  |
|  | | | | | | | | | | | | | | | | | | | | | | | |
| *Candida nivariensis*  (*Nakaseomyces nivariensis*) | | | | | | | | | | | | | | | | | | | | | | |  |
| Manogepix (6) |  |  | 1 16.7 | 2 50.0 | 3 100.0 |  |  |  |  |  |  |  |  |  |  |  |  |  |  |  |  | 0.004 |  |
| Fluconazole (6) |  |  |  |  |  |  |  |  |  |  | 0 0.0 | 2 33.3 | 2 66.7 | 1 83.3 | 0 83.3 | 0 83.3 | 0 83.3 | 1 100.0 |  |  |  | 2 |  |
| Voriconazole (6) |  |  |  |  | 0 0.0 | 2 33.3 | 1 50.0 | 2 83.3 | 0 83.3 | 0 83.3 | 0 83.3 | 1 100.0 |  |  |  |  |  |  |  |  |  | 0.03 |  |
| Anidulafungin (6) |  |  |  |  |  | 0 0.0 | 2 33.3 | 3 83.3 | 1 100.0 |  |  |  |  |  |  |  |  |  |  |  |  | 0.06 |  |
| Micafungin (6) |  |  |  | 0 0.0 | 2 33.3 | 3 83.3 | 1 100.0 |  |  |  |  |  |  |  |  |  |  |  |  |  |  | 0.015 |  |
| Amphotericin B (6) |  |  |  |  |  |  |  |  |  | 0 0.0 | 2 33.3 | 4 100.0 |  |  |  |  |  |  |  |  |  | 1 |  |
|  | | | | | | | | | | | | | | | | | | | | | | | |
| *Candida norvegensis*  (*Pichia norvegensis*) | | | | | | | | | | | | | | | | | | | | | | |  |
| Manogepix (7) |  |  |  |  |  |  |  | 0 0.0 | 1 14.3 | 0 14.3 | 3 57.1 | 3 100.0 |  |  |  |  |  |  |  |  |  | 0.5 |  |
| Fluconazole (7) |  |  |  |  |  |  |  |  |  |  |  | 0 0.0 | 1 14.3 | 0 14.3 | 1 28.6 | 3 71.4 | 2 100.0 |  |  |  |  | 16 |  |
| Voriconazole (7) |  |  |  |  |  | 0 0.0 | 1 14.3 | 0 14.3 | 2 42.9 | 2 71.4 | 2 100.0 |  |  |  |  |  |  |  |  |  |  | 0.25 |  |
| Anidulafungin (7) |  |  |  |  | 0 0.0 | 3 42.9 | 3 85.7 | 1 100.0 |  |  |  |  |  |  |  |  |  |  |  |  |  | 0.03 |  |
| Micafungin (7) |  |  |  | 0 0.0 | 1 14.3 | 0 14.3 | 2 42.9 | 4 100.0 |  |  |  |  |  |  |  |  |  |  |  |  |  | 0.06 |  |
| Amphotericin B (7) |  |  |  |  |  |  |  |  |  | 0 0.0 | 3 42.9 | 4 100.0 |  |  |  |  |  |  |  |  |  | 1 |  |
|  | | | | | | | | | | | | | | | | | | | | | | | |
| *Candida pararugosa*  (*Diutina pararugosa*) | | | | | | | | | | | | | | | | | | | | | | |  |
| Manogepix (3) |  |  | 3 100.0 |  |  |  |  |  |  |  |  |  |  |  |  |  |  |  |  |  |  | ≤0.002 |  |
| Fluconazole (3) |  |  |  |  |  |  |  |  |  |  |  |  | 0 0.0 | 3 100.0 |  |  |  |  |  |  |  | 4 |  |
| Voriconazole (3) |  |  |  |  |  |  |  | 0 0.0 | 3 100.0 |  |  |  |  |  |  |  |  |  |  |  |  | 0.12 |  |
| Anidulafungin (3) |  |  |  |  |  |  | 0 0.0 | 1 33.3 | 1 66.7 | 1 100.0 |  |  |  |  |  |  |  |  |  |  |  | 0.12 |  |
| Micafungin (3) |  |  |  |  |  |  | 0 0.0 | 1 33.3 | 2 100.0 |  |  |  |  |  |  |  |  |  |  |  |  | 0.12 |  |
| Amphotericin B (3) |  |  |  |  |  |  |  |  | 0 0.0 | 1 33.3 | 1 66.7 | 1 100.0 |  |  |  |  |  |  |  |  |  | 0.5 |  |
|  | | | | | | | | | | | | | | | | | | | | | | | |
| *Candida pseudohaemulonii* | | | | | | | | | | | | | | | | | | | | | | |  |
| Manogepix (2) |  |  | 0 0.0 | 2 100.0 |  |  |  |  |  |  |  |  |  |  |  |  |  |  |  |  |  | 0.004 |  |
| Fluconazole (2) |  |  |  |  |  |  |  |  |  |  |  |  | 0 0.0 | 1 50.0 | 0 50.0 | 1 100.0 |  |  |  |  |  | 4 |  |
| Voriconazole (2) |  |  |  |  | 0 0.0 | 1 50.0 | 0 50.0 | 0 50.0 | 0 50.0 | 0 50.0 | 1 100.0 |  |  |  |  |  |  |  |  |  |  | 0.015 |  |
| Anidulafungin (2) |  |  |  |  |  | 0 0.0 | 1 50.0 | 1 100.0 |  |  |  |  |  |  |  |  |  |  |  |  |  | 0.03 |  |
| Micafungin (2) |  |  |  |  |  | 0 0.0 | 1 50.0 | 1 100.0 |  |  |  |  |  |  |  |  |  |  |  |  |  | 0.03 |  |
| Amphotericin B (2) |  |  |  |  |  |  |  |  |  | 0 0.0 | 1 50.0 | 0 50.0 | 1 100.0 |  |  |  |  |  |  |  |  | 0.5 |  |
|  | | | | | | | | | | | | | | | | | | | | | | | |
| *Candida quercitrusa* | | | | | | | | | | | | | | | | | | | | | | |  |
| Manogepix (1) |  |  |  |  | 0 0.0 | 1 100.0 |  |  |  |  |  |  |  |  |  |  |  |  |  |  |  |  |  |
| Fluconazole (1) |  |  |  |  |  |  |  |  |  |  | 0 0.0 | 1 100.0 |  |  |  |  |  |  |  |  |  |  |  |
| Voriconazole (1) |  |  |  |  | 0 0.0 | 1 100.0 |  |  |  |  |  |  |  |  |  |  |  |  |  |  |  |  |  |
| Anidulafungin (1) |  |  |  |  |  |  |  |  |  |  |  | 0 0.0 | 1 100.0 |  |  |  |  |  |  |  |  |  |  |
| Micafungin (1) |  |  |  |  |  |  |  |  |  |  | 0 0.0 | 1 100.0 |  |  |  |  |  |  |  |  |  |  |  |
| Amphotericin B (1) |  |  |  |  |  |  |  |  | 0 0.0 | 1 100.0 |  |  |  |  |  |  |  |  |  |  |  |  |  |
|  | | | | | | | | | | | | | | | | | | | | | | | |
| *Candida rugosa* | | | | | | | | | | | | | | | | | | | | | | |  |
| Manogepix (5) |  |  | 0 0.0 | 1 20.0 | 1 40.0 | 1 60.0 | 2 100.0 |  |  |  |  |  |  |  |  |  |  |  |  |  |  | 0.015 |  |
| Fluconazole (5) |  |  |  |  |  |  |  |  |  | 0 0.0 | 1 20.0 | 0 20.0 | 2 60.0 | 1 80.0 | 1 100.0 |  |  |  |  |  |  | 2 |  |
| Voriconazole (5) |  |  |  |  | 1 20.0 | 1 40.0 | 2 80.0 | 1 100.0 |  |  |  |  |  |  |  |  |  |  |  |  |  | 0.03 |  |
| Anidulafungin (5) |  |  |  |  |  |  | 0 0.0 | 1 20.0 | 2 60.0 | 0 60.0 | 2 100.0 |  |  |  |  |  |  |  |  |  |  | 0.12 |  |
| Micafungin (5) |  |  |  |  |  |  | 0 0.0 | 4 80.0 | 1 100.0 |  |  |  |  |  |  |  |  |  |  |  |  | 0.06 |  |
| Amphotericin B (5) |  |  |  |  |  |  |  |  |  | 0 0.0 | 1 20.0 | 4 100.0 |  |  |  |  |  |  |  |  |  | 1 |  |
|  | | | | | | | | | | | | | | | | | | | | | | | |
| *Candida spencermartinsiae* | | | | | | | | | | | | | | | | | | | | | | |  |
| Manogepix (1) |  |  |  | 0 0.0 | 1 100.0 |  |  |  |  |  |  |  |  |  |  |  |  |  |  |  |  |  |  |
| Fluconazole (1) |  |  |  |  |  |  |  |  |  |  |  |  | 0 0.0 | 1 100.0 |  |  |  |  |  |  |  |  |  |
| Voriconazole (1) |  |  |  |  |  |  | 0 0.0 | 1 100.0 |  |  |  |  |  |  |  |  |  |  |  |  |  |  |  |
| Anidulafungin (1) |  |  |  |  |  |  |  |  |  | 0 0.0 | 1 100.0 |  |  |  |  |  |  |  |  |  |  |  |  |
| Micafungin (1) |  |  |  |  |  |  |  |  | 0 0.0 | 1 100.0 |  |  |  |  |  |  |  |  |  |  |  |  |  |
| Amphotericin B (1) |  |  |  |  |  |  |  |  |  | 0 0.0 | 1 100.0 |  |  |  |  |  |  |  |  |  |  |  |  |
|  | | | | | | | | | | | | | | | | | | | | | | | |
| *Candida sphaerica* | | | | | | | | | | | | | | | | | | | | | | |  |
| Manogepix (3) |  |  |  |  |  |  | 0 0.0 | 2 66.7 | 0 66.7 | 1 100.0 |  |  |  |  |  |  |  |  |  |  |  | 0.06 |  |
| Fluconazole (3) |  |  |  |  |  |  |  |  | 0 0.0 | 1 33.3 | 1 66.7 | 1 100.0 |  |  |  |  |  |  |  |  |  | 0.5 |  |
| Voriconazole (3) |  |  | 0 0.0 | 1 33.3 | 0 33.3 | 2 100.0 |  |  |  |  |  |  |  |  |  |  |  |  |  |  |  | 0.015 |  |
| Anidulafungin (3) |  |  |  |  |  | 0 0.0 | 1 33.3 | 2 100.0 |  |  |  |  |  |  |  |  |  |  |  |  |  | 0.06 |  |
| Micafungin (3) |  |  |  | 0 0.0 | 1 33.3 | 0 33.3 | 2 100.0 |  |  |  |  |  |  |  |  |  |  |  |  |  |  | 0.03 |  |
| Amphotericin B (3) |  |  |  |  |  |  |  |  |  | 0 0.0 | 2 66.7 | 1 100.0 |  |  |  |  |  |  |  |  |  | 0.5 |  |
|  | | | | | | | | | | | | | | | | | | | | | | | |
| *Candida theae* | | | | | | | | | | | | | | | | | | | | | | |  |
| Manogepix (2) |  |  | 0 0.0 | 2 100.0 |  |  |  |  |  |  |  |  |  |  |  |  |  |  |  |  |  | 0.004 |  |
| Fluconazole (2) |  |  |  |  |  |  |  |  |  | 0 0.0 | 1 50.0 | 1 100.0 |  |  |  |  |  |  |  |  |  | 0.5 |  |
| Voriconazole (2) |  |  |  | 0 0.0 | 1 50.0 | 1 100.0 |  |  |  |  |  |  |  |  |  |  |  |  |  |  |  | 0.008 |  |
| Anidulafungin (2) |  |  |  |  |  |  |  |  |  | 0 0.0 | 1 50.0 | 1 100.0 |  |  |  |  |  |  |  |  |  | 0.5 |  |
| Micafungin (2) |  |  |  |  |  |  |  |  | 0 0.0 | 2 100.0 |  |  |  |  |  |  |  |  |  |  |  | 0.25 |  |
| Amphotericin B (2) |  |  |  |  |  |  |  |  | 0 0.0 | 2 100.0 |  |  |  |  |  |  |  |  |  |  |  | 0.25 |  |
|  | | | | | | | | | | | | | | | | | | | | | | | |
| *Candida utilis* | | | | | | | | | | | | | | | | | | | | | | |  |
| Manogepix (7) |  |  | 5 71.4 | 1 85.7 | 1 100.0 |  |  |  |  |  |  |  |  |  |  |  |  |  |  |  |  | ≤0.002 |  |
| Fluconazole (7) |  |  |  |  |  |  |  |  |  | 0 0.0 | 1 14.3 | 3 57.1 | 3 100.0 |  |  |  |  |  |  |  |  | 1 |  |
| Voriconazole (7) |  |  |  |  |  | 0 0.0 | 3 42.9 | 1 57.1 | 3 100.0 |  |  |  |  |  |  |  |  |  |  |  |  | 0.06 |  |
| Anidulafungin (7) |  |  |  |  | 0 0.0 | 5 71.4 | 2 100.0 |  |  |  |  |  |  |  |  |  |  |  |  |  |  | 0.015 |  |
| Micafungin (7) |  |  |  |  | 0 0.0 | 3 42.9 | 4 100.0 |  |  |  |  |  |  |  |  |  |  |  |  |  |  | 0.03 |  |
| Amphotericin B (7) |  |  |  |  |  |  |  | 0 0.0 | 1 14.3 | 3 57.1 | 2 85.7 | 1 100.0 |  |  |  |  |  |  |  |  |  | 0.25 |  |
|  | | | | | | | | | | | | | | | | | | | | | | | |
| *Cryptococcus gattii* species complex | | | | | | | | | | | | | | | | | | | | | | |  |
| Manogepix (6) |  |  |  |  |  |  |  | 0 0.0 | 1 16.7 | 2 50.0 | 1 66.7 | 1 83.3 | 1 100.0 |  |  |  |  |  |  |  |  | 0.25 |  |
| Fluconazole (6) |  |  |  |  |  |  |  |  |  |  |  | 0 0.0 | 1 16.7 | 5 100.0 |  |  |  |  |  |  |  | 4 |  |
| Voriconazole (6) |  |  |  |  |  |  | 0 0.0 | 3 50.0 | 3 100.0 |  |  |  |  |  |  |  |  |  |  |  |  | 0.06 |  |
| Anidulafungin (6) |  |  |  |  |  |  |  |  |  |  |  |  | 0 0.0 | 3 50.0 |  |  |  |  |  | 3 100.0 |  | 4 |  |
| Micafungin (6) |  |  |  |  |  |  |  |  |  |  |  |  | 0 0.0 | 2 33.3 |  |  |  |  |  | 4 100.0 |  | >4 |  |
| Amphotericin B (6) |  |  |  |  |  |  |  |  | 0 0.0 | 1 16.7 | 3 66.7 | 2 100.0 |  |  |  |  |  |  |  |  |  | 0.5 |  |
|  | | | | | | | | | | | | | | | | | | | | | | | |
| *Cryptococcus laurentii*  (*Papiliotrema laurentii*) | | | | | | | | | | | | | | | | | | | | | | |  |
| Manogepix (1) |  |  |  |  |  |  |  |  | 0 0.0 | 1 100.0 |  |  |  |  |  |  |  |  |  |  |  |  |  |
| Fluconazole (1) |  |  |  |  |  |  |  |  |  |  |  |  |  | 0 0.0 | 1 100.0 |  |  |  |  |  |  |  |  |
| Voriconazole (1) |  |  |  |  |  |  |  |  | 0 0.0 | 1 100.0 |  |  |  |  |  |  |  |  |  |  |  |  |  |
| Anidulafungin (1) |  |  |  |  |  |  |  |  |  |  |  |  |  | 0 0.0 |  |  |  |  |  | 1 100.0 |  |  |  |
| Micafungin (1) |  |  |  |  |  |  |  |  |  |  |  |  |  | 0 0.0 |  |  |  |  |  | 1 100.0 |  |  |  |
| Amphotericin B (1) |  |  |  |  |  |  |  |  | 0 0.0 | 1 100.0 |  |  |  |  |  |  |  |  |  |  |  |  |  |
|  | | | | | | | | | | | | | | | | | | | | | | | |
| *Cryptococcus neoformans* | | | | | | | | | | | | | | | | | | | | | | |  |
| Manogepix (1) |  |  |  |  |  |  |  |  |  | 0 0.0 | 1 100.0 |  |  |  |  |  |  |  |  |  |  |  |  |
| Fluconazole (1) |  |  |  |  |  |  |  |  |  |  |  | 0 0.0 | 1 100.0 |  |  |  |  |  |  |  |  |  |  |
| Voriconazole (1) |  |  |  |  |  | 0 0.0 | 1 100.0 |  |  |  |  |  |  |  |  |  |  |  |  |  |  |  |  |
| Anidulafungin (1) |  |  |  |  |  |  |  |  |  |  |  |  |  | 0 0.0 |  |  |  |  |  | 1 100.0 |  |  |  |
| Micafungin (1) |  |  |  |  |  |  |  |  |  |  |  |  |  | 0 0.0 |  |  |  |  |  | 1 100.0 |  |  |  |
| Amphotericin B (1) |  |  |  |  |  |  |  |  |  | 0 0.0 | 1 100.0 |  |  |  |  |  |  |  |  |  |  |  |  |
|  | | | | | | | | | | | | | | | | | | | | | | | |
| *Hyphopichia burtonii* | | | | | | | | | | | | | | | | | | | | | | |  |
| Manogepix (1) | 0 0.0 | 1 100.0 |  |  |  |  |  |  |  |  |  |  |  |  |  |  |  |  |  |  |  |  |  |
| Fluconazole (1) |  |  |  |  |  |  |  |  |  | 0 0.0 | 1 100.0 |  |  |  |  |  |  |  |  |  |  |  |  |
| Voriconazole (1) |  |  |  | 0 0.0 | 1 100.0 |  |  |  |  |  |  |  |  |  |  |  |  |  |  |  |  |  |  |
| Anidulafungin (1) |  |  |  | 0 0.0 | 1 100.0 |  |  |  |  |  |  |  |  |  |  |  |  |  |  |  |  |  |  |
| Micafungin (1) |  |  |  |  |  | 0 0.0 | 1 100.0 |  |  |  |  |  |  |  |  |  |  |  |  |  |  |  |  |
| Amphotericin B (1) |  |  |  |  |  |  |  |  |  | 0 0.0 | 1 100.0 |  |  |  |  |  |  |  |  |  |  |  |  |
|  | | | | | | | | | | | | | | | | | | | | | | | |
| *Kodamaea ohmeri* | | | | | | | | | | | | | | | | | | | | | | |  |
| Manogepix (4) |  |  |  | 0 0.0 | 3 75.0 | 1 100.0 |  |  |  |  |  |  |  |  |  |  |  |  |  |  |  | 0.008 |  |
| Fluconazole (4) |  |  |  |  |  |  |  |  |  |  |  |  | 0 0.0 | 3 75.0 | 0 75.0 | 1 100.0 |  |  |  |  |  | 4 |  |
| Voriconazole (4) |  |  |  |  |  | 0 0.0 | 3 75.0 | 0 75.0 | 1 100.0 |  |  |  |  |  |  |  |  |  |  |  |  | 0.03 |  |
| Anidulafungin (4) |  |  |  |  |  |  |  | 0 0.0 | 2 50.0 | 0 50.0 | 0 50.0 | 1 75.0 | 0 75.0 | 0 75.0 |  |  |  |  |  | 1 100.0 |  | 0.12 |  |
| Micafungin (4) |  |  |  |  |  |  | 0 0.0 | 1 25.0 | 1 50.0 | 0 50.0 | 1 75.0 | 0 75.0 | 0 75.0 | 0 75.0 |  |  |  |  |  | 1 100.0 |  | 0.12 |  |
| Amphotericin B (4) |  |  |  |  |  |  |  |  | 0 0.0 | 2 50.0 | 2 100.0 |  |  |  |  |  |  |  |  |  |  | 0.25 |  |
|  | | | | | | | | | | | | | | | | | | | | | | | |
| *Lodderomyces elongisporus* | | | | | | | | | | | | | | | | | | | | | | |  |
| Manogepix (1) |  |  | 0 0.0 | 1 100.0 |  |  |  |  |  |  |  |  |  |  |  |  |  |  |  |  |  |  |  |
| Fluconazole (1) |  |  |  |  |  |  |  |  |  | 0 0.0 | 1 100.0 |  |  |  |  |  |  |  |  |  |  |  |  |
| Voriconazole (1) |  |  |  |  | 0 0.0 | 1 100.0 |  |  |  |  |  |  |  |  |  |  |  |  |  |  |  |  |  |
| Anidulafungin (1) |  |  |  |  | 0 0.0 | 1 100.0 |  |  |  |  |  |  |  |  |  |  |  |  |  |  |  |  |  |
| Micafungin (1) |  |  |  |  |  | 0 0.0 | 1 100.0 |  |  |  |  |  |  |  |  |  |  |  |  |  |  |  |  |
| Amphotericin B (1) |  |  |  |  |  |  |  |  | 0 0.0 | 1 100.0 |  |  |  |  |  |  |  |  |  |  |  |  |  |
|  | | | | | | | | | | | | | | | | | | | | | | | |
| *Saprochaete capitata* | | | | | | | | | | | | | | | | | | | | | | |  |
| Manogepix (4) |  |  |  |  | 0 0.0 | 2 50.0 | 1 75.0 | 1 100.0 |  |  |  |  |  |  |  |  |  |  |  |  |  | 0.015 |  |
| Fluconazole (4) |  |  |  |  |  |  |  |  |  |  | 0 0.0 | 1 25.0 | 0 25.0 | 0 25.0 | 3 100.0 |  |  |  |  |  |  | 8 |  |
| Voriconazole (4) |  |  |  |  |  |  | 0 0.0 | 1 25.0 | 1 50.0 | 2 100.0 |  |  |  |  |  |  |  |  |  |  |  | 0.12 |  |
| Anidulafungin (4) |  |  |  |  |  |  |  |  |  |  |  | 0 0.0 | 1 25.0 | 3 100.0 |  |  |  |  |  |  |  | 4 |  |
| Micafungin (4) |  |  |  |  |  |  |  |  |  |  |  | 0 0.0 | 1 25.0 | 1 50.0 |  |  |  |  |  | 2 100.0 |  | 4 |  |
| Amphotericin B (4) |  |  |  |  |  |  |  |  |  |  | 0 0.0 | 4 100.0 |  |  |  |  |  |  |  |  |  | 1 |  |
|  | | | | | | | | | | | | | | | | | | | | | | | |
| *Ogataea siamensis* | | | | | | | | | | | | | | | | | | | | | | |  |
| Manogepix (1) |  |  |  |  |  | 0 0.0 | 1 100.0 |  |  |  |  |  |  |  |  |  |  |  |  |  |  |  |  |
| Fluconazole (1) |  |  |  |  |  |  |  |  | 0 0.0 | 1 100.0 |  |  |  |  |  |  |  |  |  |  |  |  |  |
| Voriconazole (1) |  |  |  |  | 1 100.0 |  |  |  |  |  |  |  |  |  |  |  |  |  |  |  |  |  |  |
| Anidulafungin (1) |  |  |  |  |  | 0 0.0 | 1 100.0 |  |  |  |  |  |  |  |  |  |  |  |  |  |  |  |  |
| Micafungin (1) |  |  |  |  |  | 0 0.0 | 1 100.0 |  |  |  |  |  |  |  |  |  |  |  |  |  |  |  |  |
| Amphotericin B (1) |  |  |  |  |  |  |  |  | 0 0.0 | 1 100.0 |  |  |  |  |  |  |  |  |  |  |  |  |  |
|  | | | | | | | | | | | | | | | | | | | | | | | |
| *Pichia cactophila* | | | | | | | | | | | | | | | | | | | | | | |  |
| Manogepix (2) |  |  |  |  |  |  |  |  |  | 0 0.0 | 1 50.0 | 1 100.0 |  |  |  |  |  |  |  |  |  | 0.5 |  |
| Fluconazole (2) |  |  |  |  |  |  |  |  |  |  |  |  |  | 0 0.0 | 1 50.0 | 1 100.0 |  |  |  |  |  | 8 |  |
| Voriconazole (2) |  |  |  |  |  | 0 0.0 | 1 50.0 | 0 50.0 | 1 100.0 |  |  |  |  |  |  |  |  |  |  |  |  | 0.03 |  |
| Anidulafungin (2) |  |  |  |  |  | 0 0.0 | 2 100.0 |  |  |  |  |  |  |  |  |  |  |  |  |  |  | 0.03 |  |
| Micafungin (2) |  |  |  |  |  | 0 0.0 | 2 100.0 |  |  |  |  |  |  |  |  |  |  |  |  |  |  | 0.03 |  |
| Amphotericin B (2) |  |  |  |  |  |  |  |  | 0 0.0 | 1 50.0 | 1 100.0 |  |  |  |  |  |  |  |  |  |  | 0.25 |  |
|  | | | | | | | | | | | | | | | | | | | | | | | |
| *Pichia kluyveri* | | | | | | | | | | | | | | | | | | | | | | |  |
| Manogepix (1) |  |  |  |  |  |  | 0 0.0 | 1 100.0 |  |  |  |  |  |  |  |  |  |  |  |  |  |  |  |
| Fluconazole (1) |  |  |  |  |  |  |  |  |  |  |  |  |  |  |  |  | 0 0.0 | 1 100.0 |  |  |  |  |  |
| Voriconazole (1) |  |  |  |  |  |  |  | 0 0.0 | 1 100.0 |  |  |  |  |  |  |  |  |  |  |  |  |  |  |
| Anidulafungin (1) |  |  |  | 0 0.0 | 1 100.0 |  |  |  |  |  |  |  |  |  |  |  |  |  |  |  |  |  |  |
| Micafungin (1) |  |  |  |  | 0 0.0 | 1 100.0 |  |  |  |  |  |  |  |  |  |  |  |  |  |  |  |  |  |
| Amphotericin B (1) |  |  |  |  |  |  |  |  |  | 0 0.0 | 1 100.0 |  |  |  |  |  |  |  |  |  |  |  |  |
|  | | | | | | | | | | | | | | | | | | | | | | | |
| *Rhodotorula minuta* | | | | | | | | | | | | | | | | | | | | | | |  |
| Manogepix (1) |  |  |  |  | 0 0.0 | 1 100.0 |  |  |  |  |  |  |  |  |  |  |  |  |  |  |  |  |  |
| Fluconazole (1) |  |  |  |  |  |  |  |  |  |  |  |  |  |  |  |  |  |  | 0 0.0 | 1 100.0 |  |  |  |
| Voriconazole (1) |  |  |  |  |  |  |  |  |  |  | 0 0.0 | 1 100.0 |  |  |  |  |  |  |  |  |  |  |  |
| Anidulafungin (1) |  |  |  |  |  |  |  |  |  |  |  |  |  | 0 0.0 |  |  |  |  |  | 1 100.0 |  |  |  |
| Micafungin (1) |  |  |  |  |  |  |  |  |  |  |  |  | 0 0.0 | 1 100.0 |  |  |  |  |  |  |  |  |  |
| Amphotericin B (1) |  |  |  |  |  |  |  |  |  |  | 0 0.0 | 1 100.0 |  |  |  |  |  |  |  |  |  |  |  |
|  | | | | | | | | | | | | | | | | | | | | | | | |
| *Trichosporon capitatum* | | | | | | | | | | | | | | | | | | | | | | |  |
| Manogepix (1) |  |  |  |  |  | 0 0.0 | 1 100.0 |  |  |  |  |  |  |  |  |  |  |  |  |  |  |  |  |
| Fluconazole (1) |  |  |  |  |  |  |  |  |  |  |  |  |  | 0 0.0 | 1 100.0 |  |  |  |  |  |  |  |  |
| Voriconazole (1) |  |  |  |  |  |  |  |  | 0 0.0 | 1 100.0 |  |  |  |  |  |  |  |  |  |  |  |  |  |
| Anidulafungin (1) |  |  |  |  |  |  |  |  |  |  |  | 0 0.0 | 1 100.0 |  |  |  |  |  |  |  |  |  |  |
| Micafungin (1) |  |  |  |  |  |  |  |  |  |  |  | 0 0.0 | 1 100.0 |  |  |  |  |  |  |  |  |  |  |
| Amphotericin B (1) |  |  |  |  |  |  |  |  |  |  | 0 0.0 | 1 100.0 |  |  |  |  |  |  |  |  |  |  |  |
|  | | | | | | | | | | | | | | | | | | | | | | | |
| *Trichosporon inkin* | | | | | | | | | | | | | | | | | | | | | | |  |
| Manogepix (2) |  |  |  |  |  |  |  |  |  |  | 0 0.0 | 1 50.0 | 1 100.0 |  |  |  |  |  |  |  |  | 1 |  |
| Fluconazole (2) |  |  |  |  |  |  |  |  | 0 0.0 | 1 50.0 | 1 100.0 |  |  |  |  |  |  |  |  |  |  | 0.25 |  |
| Voriconazole (2) |  |  |  | 0 0.0 | 1 50.0 | 1 100.0 |  |  |  |  |  |  |  |  |  |  |  |  |  |  |  | 0.008 |  |
| Anidulafungin (2) |  |  |  |  |  |  |  |  |  |  |  |  |  | 0 0.0 |  |  |  |  |  | 2 100.0 |  | >4 |  |
| Micafungin (2) |  |  |  |  |  |  |  |  |  |  |  |  |  | 0 0.0 |  |  |  |  |  | 2 100.0 |  | >4 |  |
| Amphotericin B (2) |  |  |  |  |  |  |  |  |  | 0 0.0 | 2 100.0 |  |  |  |  |  |  |  |  |  |  | 0.5 |  |
|  | | | | | | | | | | | | | | | | | | | | | | | |
| *Trichosporon loubieri (Apiotrichum loubieri*) | | | | | | | | | | | | | | | | | | | | | | |  |
| Manogepix (1) |  |  |  |  |  |  |  |  |  | 0 0.0 | 1 100.0 |  |  |  |  |  |  |  |  |  |  |  |  |
| Fluconazole (1) |  |  |  |  |  |  |  |  |  | 0 0.0 | 1 100.0 |  |  |  |  |  |  |  |  |  |  |  |  |
| Voriconazole (1) |  |  |  | 0 0.0 | 1 100.0 |  |  |  |  |  |  |  |  |  |  |  |  |  |  |  |  |  |  |
| Anidulafungin (1) |  |  |  |  |  |  |  |  |  |  |  |  | 0 0.0 | 1 100.0 |  |  |  |  |  |  |  |  |  |
| Micafungin (1) |  |  |  |  |  |  |  |  |  |  |  |  |  | 0 0.0 |  |  |  |  |  | 1 100.0 |  |  |  |
| Amphotericin B (1) |  |  |  |  |  |  |  |  |  |  | 0 0.0 | 1 100.0 |  |  |  |  |  |  |  |  |  |  |  |
|  | | | | | | | | | | | | | | | | | | | | | | | |
| *Trichosporon mucoides*  (*Cutaneotrichosporon mucoides*) | | | | | | | | | | | | | | | | | | | | | | |  |
| Manogepix (2) |  |  |  |  |  |  |  |  |  |  |  |  | 0 0.0 |  |  |  |  |  |  | 2 100.0 |  | >2 |  |
| Fluconazole (2) |  |  |  |  |  |  |  |  |  |  |  |  | 0 0.0 | 1 50.0 | 0 50.0 | 0 50.0 | 0 50.0 | 0 50.0 | 0 50.0 | 1 100.0 |  | 4 |  |
| Voriconazole (2) |  |  |  |  |  |  | 0 0.0 | 1 50.0 | 0 50.0 | 0 50.0 | 0 50.0 | 0 50.0 | 0 50.0 | 0 50.0 | 0 50.0 |  |  |  |  | 1 100.0 |  | 0.06 |  |
| Anidulafungin (2) |  |  |  |  |  |  |  |  |  |  |  |  |  | 0 0.0 |  |  |  |  |  | 2 100.0 |  | >4 |  |
| Micafungin (2) |  |  |  |  |  |  |  |  |  |  |  |  |  | 0 0.0 |  |  |  |  |  | 2 100.0 |  | >4 |  |
| Amphotericin B (2) |  |  |  |  |  |  |  |  |  | 0 0.0 | 2 100.0 |  |  |  |  |  |  |  |  |  |  | 0.5 |  |

^a^ Greater than the highest concentration tested.
