# Supplementary material for: In vitro activity of manogepix and comparators against infrequently encountered yeast and mold isolates from the SENTRY Surveillance Program (2017–2022)
Source: Antimicrob Agents Chemother. 2024 Jan 11;68(2):e01132-23. doi: 10.1128/aac.01132-23 (PMC10848754; doi:10.1128/aac.01132-23)
Supplement: Table S2 — Antimicrobial activity of manogepix and comparators against infrequently encountered mold isolates. [file aac.01132-23-s0002.docx]

Table S2 Antimicrobial activity of manogepix and comparator against infrequently encountered mold isolates

| **Organism/organism group (no. of isolates)** | **No. and cumulative % of isolates inhibited at MIC (mg/L) of:** | | | | | | | | | | | | | | |  | **MIC_50_** | **MIC_90_** |
| --- | --- | --- | --- | --- | --- | --- | --- | --- | --- | --- | --- | --- | --- | --- | --- | --- | --- | --- |
|  | **≤0.001** | **0.002** | **0.004** | **0.008** | **0.015** | **0.03** | **0.06** | **0.12** | **0.25** | **0.5** | **1** | **2** | **4** | **8** | **> ^a^** |  |  |  |
| *Alternaria alternata* | | | | | | | | | | | | | | | | | |  |
| Manogepix (1) |  |  |  |  |  |  |  |  |  | 0 0.0 | 1 100.0 |  |  |  |  |  |  |  |
| Voriconazole (1) |  |  |  |  |  |  |  |  |  |  | 0 0.0 | 1 100.0 |  |  |  |  |  |  |
| Anidulafungin (1) |  |  |  |  |  |  | 0 0.0 | 1 100.0 |  |  |  |  |  |  |  |  |  |  |
| Micafungin (1) |  |  |  |  | 0 0.0 | 1 100.0 |  |  |  |  |  |  |  |  |  |  |  |  |
| Itraconazole (1) |  |  |  |  |  |  |  | 0 0.0 | 1 100.0 |  |  |  |  |  |  |  |  |  |
| Amphotericin B (1) |  |  |  |  |  |  |  |  |  | 0 0.0 | 1 100.0 |  |  |  |  |  |  |  |
|  | | | | | | | | | | | | | | | | | | |
| *Aspergillus alabamensis* | | | | | | | | | | | | | | | | | |  |
| Manogepix (1) |  |  | 0 0.0 | 1 100.0 |  |  |  |  |  |  |  |  |  |  |  |  |  |  |
| Voriconazole (1) |  |  |  |  |  |  |  |  | 0 0.0 | 1 100.0 |  |  |  |  |  |  |  |  |
| Anidulafungin (1) |  |  |  |  | 0 0.0 | 1 100.0 |  |  |  |  |  |  |  |  |  |  |  |  |
| Micafungin (1) |  |  | 0 0.0 | 1 100.0 |  |  |  |  |  |  |  |  |  |  |  |  |  |  |
| Itraconazole (1) |  |  |  |  |  |  |  |  | 0 0.0 | 1 100.0 |  |  |  |  |  |  |  |  |
| Amphotericin B (1) |  |  |  |  |  |  |  |  |  | 0 0.0 | 1 100.0 |  |  |  |  |  |  |  |
|  | | | | | | | | | | | | | | | | | | |
| *Aspergillus brasiliensis* | | | | | | | | | | | | | | | | | |  |
| Manogepix (2) |  |  | 0 0.0 | 1 50.0 | 1 100.0 |  |  |  |  |  |  |  |  |  |  |  | 0.008 |  |
| Voriconazole (2) |  |  |  |  |  |  |  |  | 0 0.0 | 1 50.0 | 0 50.0 | 0 50.0 | 0 50.0 | 1 100.0 |  |  | 0.5 |  |
| Anidulafungin (2) |  | 0 0.0 | 1 50.0 | 1 100.0 |  |  |  |  |  |  |  |  |  |  |  |  | 0.004 |  |
| Micafungin (2) |  | 1 50.0 | 0 50.0 | 0 50.0 | 1 100.0 |  |  |  |  |  |  |  |  |  |  |  | ≤0.002 |  |
| Itraconazole (2) |  |  |  |  |  |  |  |  |  | 0 0.0 | 1 50.0 | 0 50.0 | 0 50.0 | 0 50.0 | 1 100.0 |  | 1 |  |
| Amphotericin B (2) |  |  |  |  |  |  |  | 0 0.0 | 2 100.0 |  |  |  |  |  |  |  | 0.25 |  |
|  | | | | | | | | | | | | | | | | | | |
| *Aspergillus clavatus* | | | | | | | | | | | | | | | | | |  |
| Manogepix (2) |  |  |  |  | 0 0.0 | 2 100.0 |  |  |  |  |  |  |  |  |  |  | 0.03 |  |
| Voriconazole (2) |  |  |  |  |  |  |  |  |  |  |  | 0 0.0 | 2 100.0 |  |  |  | 4 |  |
| Anidulafungin (2) |  |  |  | 0 0.0 | 1 50.0 | 0 50.0 | 1 100.0 |  |  |  |  |  |  |  |  |  | 0.015 |  |
| Micafungin (2) |  | 1 50.0 | 0 50.0 | 0 50.0 | 0 50.0 | 0 50.0 | 0 50.0 | 1 100.0 |  |  |  |  |  |  |  |  | ≤0.002 |  |
| Itraconazole (2) |  |  |  |  |  |  |  |  |  |  |  |  |  | 0 0.0 | 2 100.0 |  | >8 |  |
| Amphotericin B (2) |  |  |  |  |  | 0 0.0 | 1 50.0 | 1 100.0 |  |  |  |  |  |  |  |  | 0.06 |  |
|  | | | | | | | | | | | | | | | | | | |
| *Aspergillus flavus* | | | | | | | | | | | | | | | | | |  |
| Manogepix (7) |  |  |  | 0 0.0 | 1 14.3 | 4 71.4 | 2 100.0 |  |  |  |  |  |  |  |  |  | 0.03 |  |
| Voriconazole (7) |  |  |  |  |  |  |  |  | 0 0.0 | 4 57.1 | 3 100.0 |  |  |  |  |  | 0.5 |  |
| Anidulafungin (7) |  | 0 0.0 | 1 14.3 | 5 85.7 | 1 100.0 |  |  |  |  |  |  |  |  |  |  |  | 0.008 |  |
| Micafungin (7) |  |  | 0 0.0 | 2 28.6 | 5 100.0 |  |  |  |  |  |  |  |  |  |  |  | 0.015 |  |
| Itraconazole (7) |  |  |  |  |  |  |  |  | 0 0.0 | 6 85.7 | 1 100.0 |  |  |  |  |  | 0.5 |  |
| Amphotericin B (7) |  |  |  |  |  |  |  |  | 0 0.0 | 4 57.1 | 3 100.0 |  |  |  |  |  | 0.5 |  |
|  | | | | | | | | | | | | | | | | | | |
| *Aspergillus fumisynnematus* | | | | | | | | | | | | | | | | | |  |
| Manogepix (1) |  |  | 0 0.0 | 1 100.0 |  |  |  |  |  |  |  |  |  |  |  |  |  |  |
| Voriconazole (1) |  |  |  |  |  |  |  |  |  |  |  | 0 0.0 | 1 100.0 |  |  |  |  |  |
| Anidulafungin (1) |  |  | 0 0.0 | 1 100.0 |  |  |  |  |  |  |  |  |  |  |  |  |  |  |
| Micafungin (1) |  |  | 0 0.0 | 1 100.0 |  |  |  |  |  |  |  |  |  |  |  |  |  |  |
| Itraconazole (1) |  |  |  |  |  |  |  |  |  | 0 0.0 | 1 100.0 |  |  |  |  |  |  |  |
| Amphotericin B (1) |  |  |  |  |  |  |  |  |  | 0 0.0 | 1 100.0 |  |  |  |  |  |  |  |
|  | | | | | | | | | | | | | | | | | | |
| *Aspergillus hortai* | | | | | | | | | | | | | | | | | |  |
| Manogepix (1) |  |  | 0 0.0 | 1 100.0 |  |  |  |  |  |  |  |  |  |  |  |  |  |  |
| Voriconazole (1) |  |  |  |  |  |  | 0 0.0 | 1 100.0 |  |  |  |  |  |  |  |  |  |  |
| Anidulafungin (1) |  |  |  |  |  | 0 0.0 | 1 100.0 |  |  |  |  |  |  |  |  |  |  |  |
| Micafungin (1) |  |  | 0 0.0 | 1 100.0 |  |  |  |  |  |  |  |  |  |  |  |  |  |  |
| Itraconazole (1) |  |  |  |  |  |  |  |  | 0 0.0 | 1 100.0 |  |  |  |  |  |  |  |  |
| Amphotericin B (1) |  |  |  |  |  |  |  |  |  |  |  | 0 0.0 | 1 100.0 |  |  |  |  |  |
|  | | | | | | | | | | | | | | | | | | |
| *Aspergillus lentulus* | | | | | | | | | | | | | | | | | |  |
| Manogepix (7) |  |  | 0 0.0 | 3 42.9 | 4 100.0 |  |  |  |  |  |  |  |  |  |  |  | 0.015 |  |
| Voriconazole (7) |  |  |  |  |  |  |  |  |  |  | 0 0.0 | 7 100.0 |  |  |  |  | 2 |  |
| Anidulafungin (7) |  |  |  | 0 0.0 | 2 28.6 | 3 71.4 | 1 85.7 | 1 100.0 |  |  |  |  |  |  |  |  | 0.03 |  |
| Micafungin (7) |  |  | 0 0.0 | 3 42.9 | 4 100.0 |  |  |  |  |  |  |  |  |  |  |  | 0.015 |  |
| Itraconazole (7) |  |  |  |  |  |  |  |  |  | 0 0.0 | 3 42.9 | 4 100.0 |  |  |  |  | 2 |  |
| Amphotericin B (7) |  |  |  |  |  |  |  |  |  |  | 0 0.0 | 6 85.7 | 0 85.7 |  | 1 100.0 |  | 2 |  |
|  | | | | | | | | | | | | | | | | | | |
| *Aspergillus melleus* | | | | | | | | | | | | | | | | | |  |
| Manogepix (1) |  |  |  | 0 0.0 | 1 100.0 |  |  |  |  |  |  |  |  |  |  |  |  |  |
| Voriconazole (1) |  |  |  |  |  |  |  | 0 0.0 | 1 100.0 |  |  |  |  |  |  |  |  |  |
| Anidulafungin (1) |  |  |  |  |  | 0 0.0 | 1 100.0 |  |  |  |  |  |  |  |  |  |  |  |
| Micafungin (1) |  |  |  |  | 0 0.0 | 1 100.0 |  |  |  |  |  |  |  |  |  |  |  |  |
| Itraconazole (1) |  |  |  |  |  |  |  |  | 0 0.0 | 1 100.0 |  |  |  |  |  |  |  |  |
| Amphotericin B (1) |  |  |  |  |  |  |  |  |  |  | 0 0.0 | 1 100.0 |  |  |  |  |  |  |
|  | | | | | | | | | | | | | | | | | | |
| *Aspergillus nomius* | | | | | | | | | | | | | | | | | |  |
| Manogepix (1) |  |  | 0 0.0 | 1 100.0 |  |  |  |  |  |  |  |  |  |  |  |  |  |  |
| Voriconazole (1) |  |  |  |  |  |  |  |  | 0 0.0 | 1 100.0 |  |  |  |  |  |  |  |  |
| Anidulafungin (1) |  | 0 0.0 | 1 100.0 |  |  |  |  |  |  |  |  |  |  |  |  |  |  |  |
| Micafungin (1) |  |  | 0 0.0 | 1 100.0 |  |  |  |  |  |  |  |  |  |  |  |  |  |  |
| Itraconazole (1) |  |  |  |  |  |  |  |  | 0 0.0 | 1 100.0 |  |  |  |  |  |  |  |  |
| Amphotericin B (1) |  |  |  |  |  |  |  |  |  |  | 0 0.0 | 1 100.0 |  |  |  |  |  |  |
|  | | | | | | | | | | | | | | | | | | |
| *Aspergillus ochraceus* species complex | | | | | | | | | | | | | | | | | |  |
| Manogepix (1) |  |  |  |  |  |  | 0 0.0 | 1 100.0 |  |  |  |  |  |  |  |  |  |  |
| Voriconazole (1) |  |  |  |  |  |  |  |  | 0 0.0 | 1 100.0 |  |  |  |  |  |  |  |  |
| Anidulafungin (1) |  |  |  |  |  |  | 0 0.0 | 1 100.0 |  |  |  |  |  |  |  |  |  |  |
| Micafungin (1) |  |  |  |  |  | 0 0.0 | 1 100.0 |  |  |  |  |  |  |  |  |  |  |  |
| Itraconazole (1) |  |  |  |  |  |  |  |  |  |  | 0 0.0 | 1 100.0 |  |  |  |  |  |  |
| Amphotericin B (1) |  |  |  |  |  |  |  |  |  |  |  |  | 0 0.0 |  | 1 100.0 |  |  |  |
|  | | | | | | | | | | | | | | | | | | |
| *Aspergillus parasiticus* | | | | | | | | | | | | | | | | | |  |
| Manogepix (3) |  |  | 0 0.0 | 2 66.7 | 1 100.0 |  |  |  |  |  |  |  |  |  |  |  | 0.008 |  |
| Voriconazole (3) |  |  |  |  |  |  |  | 0 0.0 | 1 33.3 | 1 66.7 | 1 100.0 |  |  |  |  |  | 0.5 |  |
| Anidulafungin (3) |  |  | 0 0.0 | 3 100.0 |  |  |  |  |  |  |  |  |  |  |  |  | 0.008 |  |
| Micafungin (3) |  | 2 66.7 | 0 66.7 | 1 100.0 |  |  |  |  |  |  |  |  |  |  |  |  | ≤0.002 |  |
| Itraconazole (3) |  |  |  |  |  |  |  |  | 0 0.0 | 1 33.3 | 2 100.0 |  |  |  |  |  | 1 |  |
| Amphotericin B (3) |  |  |  |  |  |  |  |  |  |  | 0 0.0 | 3 100.0 |  |  |  |  | 2 |  |
|  | | | | | | | | | | | | | | | | | | |
| *Aspergillus sclerotiorum* | | | | | | | | | | | | | | | | | |  |
| Manogepix (3) |  |  |  | 0 0.0 | 2 66.7 | 1 100.0 |  |  |  |  |  |  |  |  |  |  | 0.015 |  |
| Voriconazole (3) |  |  |  |  |  |  | 0 0.0 | 1 33.3 | 0 33.3 | 1 66.7 | 1 100.0 |  |  |  |  |  | 0.5 |  |
| Anidulafungin (3) |  |  |  |  | 0 0.0 | 1 33.3 | 1 66.7 | 0 66.7 | 0 66.7 | 1 100.0 |  |  |  |  |  |  | 0.06 |  |
| Micafungin (3) |  |  |  |  | 0 0.0 | 2 66.7 | 0 66.7 | 1 100.0 |  |  |  |  |  |  |  |  | 0.03 |  |
| Itraconazole (3) |  |  |  |  |  |  |  |  |  | 0 0.0 | 2 66.7 | 0 66.7 | 0 66.7 | 0 66.7 | 1 100.0 |  | 1 |  |
| Amphotericin B (3) |  |  |  |  |  |  |  |  |  | 0 0.0 | 1 33.3 | 1 66.7 | 0 66.7 |  | 1 100.0 |  | 2 |  |
|  | | | | | | | | | | | | | | | | | | |
| *Aspergillus sydowii* | | | | | | | | | | | | | | | | | |  |
| Manogepix (4) | 0 0.0 | 1 25.0 | 0 25.0 | 0 25.0 | 3 100.0 |  |  |  |  |  |  |  |  |  |  |  | 0.015 |  |
| Voriconazole (4) |  |  |  |  |  |  |  | 0 0.0 | 1 25.0 | 3 100.0 |  |  |  |  |  |  | 0.5 |  |
| Anidulafungin (3) |  |  | 0 0.0 | 2 66.7 | 0 66.7 | 0 66.7 | 1 100.0 |  |  |  |  |  |  |  |  |  | 0.008 |  |
| Micafungin (3) |  | 0 0.0 | 2 66.7 | 0 66.7 | 0 66.7 | 1 100.0 |  |  |  |  |  |  |  |  |  |  | 0.004 |  |
| Itraconazole (4) |  |  |  |  |  |  |  |  | 0 0.0 | 2 50.0 | 2 100.0 |  |  |  |  |  | 0.5 |  |
| Amphotericin B (4) |  |  |  |  |  |  |  |  |  | 0 0.0 | 3 75.0 | 1 100.0 |  |  |  |  | 1 |  |
|  | | | | | | | | | | | | | | | | | | |
| *Aspergillus tamarii* | | | | | | | | | | | | | | | | | |  |
| Manogepix (3) |  |  |  |  | 0 0.0 | 2 66.7 | 1 100.0 |  |  |  |  |  |  |  |  |  | 0.03 |  |
| Voriconazole (3) |  |  |  |  |  |  |  | 0 0.0 | 1 33.3 | 2 100.0 |  |  |  |  |  |  | 0.5 |  |
| Anidulafungin (3) |  | 0 0.0 | 2 66.7 | 0 66.7 | 1 100.0 |  |  |  |  |  |  |  |  |  |  |  | 0.004 |  |
| Micafungin (3) |  | 1 33.3 | 0 33.3 | 1 66.7 | 1 100.0 |  |  |  |  |  |  |  |  |  |  |  | 0.008 |  |
| Itraconazole (3) |  |  |  |  |  |  |  | 0 0.0 | 2 66.7 | 1 100.0 |  |  |  |  |  |  | 0.25 |  |
| Amphotericin B (3) |  |  |  |  |  |  |  |  |  | 0 0.0 | 1 33.3 | 2 100.0 |  |  |  |  | 2 |  |
|  | | | | | | | | | | | | | | | | | | |
| *Aspergillus thermomutatus* | | | | | | | | | | | | | | | | | |  |
| Manogepix (2) |  |  |  |  |  | 0 0.0 | 1 50.0 | 0 50.0 | 1 100.0 |  |  |  |  |  |  |  | 0.06 |  |
| Voriconazole (2) |  |  |  |  |  |  |  |  |  |  |  | 0 0.0 | 1 50.0 | 1 100.0 |  |  | 4 |  |
| Anidulafungin (2) |  |  |  | 0 0.0 | 1 50.0 | 1 100.0 |  |  |  |  |  |  |  |  |  |  | 0.015 |  |
| Micafungin (2) |  |  |  |  | 0 0.0 | 2 100.0 |  |  |  |  |  |  |  |  |  |  | 0.03 |  |
| Itraconazole (2) |  |  |  |  |  |  |  |  |  |  |  | 0 0.0 | 2 100.0 |  |  |  | 4 |  |
| Amphotericin B (2) |  |  |  |  |  |  |  |  | 0 0.0 | 1 50.0 | 1 100.0 |  |  |  |  |  | 0.5 |  |
|  | | | | | | | | | | | | | | | | | | |
| *Aspergillus udagawae* | | | | | | | | | | | | | | | | | |  |
| Manogepix (2) |  |  |  | 0 0.0 | 2 100.0 |  |  |  |  |  |  |  |  |  |  |  | 0.015 |  |
| Voriconazole (2) |  |  |  |  |  |  |  |  |  |  | 0 0.0 | 2 100.0 |  |  |  |  | 2 |  |
| Anidulafungin (2) |  |  |  | 0 0.0 | 1 50.0 | 0 50.0 | 1 100.0 |  |  |  |  |  |  |  |  |  | 0.015 |  |
| Micafungin (2) |  |  | 0 0.0 | 2 100.0 |  |  |  |  |  |  |  |  |  |  |  |  | 0.008 |  |
| Itraconazole (2) |  |  |  |  |  |  |  |  |  |  | 0 0.0 | 1 50.0 | 1 100.0 |  |  |  | 2 |  |
| Amphotericin B (2) |  |  |  |  |  |  |  |  |  | 0 0.0 | 1 50.0 | 1 100.0 |  |  |  |  | 1 |  |
|  | | | | | | | | | | | | | | | | | | |
| *Aspergillus unguis* | | | | | | | | | | | | | | | | | |  |
| Manogepix (3) |  |  |  |  | 0 0.0 | 3 100.0 |  |  |  |  |  |  |  |  |  |  | 0.03 |  |
| Voriconazole (3) |  |  |  |  |  | 0 0.0 | 1 33.3 | 0 33.3 | 1 66.7 | 1 100.0 |  |  |  |  |  |  | 0.25 |  |
| Anidulafungin (3) |  | 1 33.3 | 0 33.3 | 0 33.3 | 2 100.0 |  |  |  |  |  |  |  |  |  |  |  | 0.015 |  |
| Micafungin (3) |  | 0 0.0 | 2 66.7 | 0 66.7 | 1 100.0 |  |  |  |  |  |  |  |  |  |  |  | 0.004 |  |
| Itraconazole (3) |  |  |  |  |  |  |  |  | 0 0.0 | 1 33.3 | 1 66.7 | 0 66.7 | 0 66.7 | 0 66.7 | 1 100.0 |  | 1 |  |
| Amphotericin B (3) |  |  |  |  |  |  |  |  |  | 0 0.0 | 1 33.3 | 1 66.7 | 1 100.0 |  |  |  | 2 |  |
|  | | | | | | | | | | | | | | | | | | |
| *Aspergillus ustus* | | | | | | | | | | | | | | | | | |  |
| Manogepix (3) |  |  |  | 2 66.7 | 1 100.0 |  |  |  |  |  |  |  |  |  |  |  | ≤0.008 |  |
| Voriconazole (3) |  |  |  |  |  |  |  |  |  |  |  | 0 0.0 | 2 66.7 | 1 100.0 |  |  | 4 |  |
| Anidulafungin (3) |  |  |  |  | 0 0.0 | 1 33.3 | 0 33.3 | 2 100.0 |  |  |  |  |  |  |  |  | 0.12 |  |
| Micafungin (3) |  |  |  | 0 0.0 | 1 33.3 | 1 66.7 | 1 100.0 |  |  |  |  |  |  |  |  |  | 0.03 |  |
| Itraconazole (3) |  |  |  |  |  |  |  |  |  |  |  | 0 0.0 | 1 33.3 | 2 100.0 |  |  | 8 |  |
| Amphotericin B (3) |  |  |  |  |  |  |  |  |  | 0 0.0 | 3 100.0 |  |  |  |  |  | 1 |  |
|  | | | | | | | | | | | | | | | | | | |
| *Aspergillus versicolor* | | | | | | | | | | | | | | | | | |  |
| Manogepix (7) |  | 1 14.3 | 0 14.3 | 1 28.6 | 4 85.7 | 1 100.0 |  |  |  |  |  |  |  |  |  |  | 0.015 |  |
| Voriconazole (7) |  |  |  |  |  |  |  | 0 0.0 | 1 14.3 | 3 57.1 | 3 100.0 |  |  |  |  |  | 0.5 |  |
| Anidulafungin (7) |  |  |  | 0 0.0 | 4 57.1 | 2 85.7 | 1 100.0 |  |  |  |  |  |  |  |  |  | 0.015 |  |
| Micafungin (7) |  | 0 0.0 | 1 14.3 | 2 42.9 | 3 85.7 | 1 100.0 |  |  |  |  |  |  |  |  |  |  | 0.015 |  |
| Itraconazole (7) |  |  |  |  |  |  |  |  | 0 0.0 | 2 28.6 | 3 71.4 | 2 100.0 |  |  |  |  | 1 |  |
| Amphotericin B (7) |  |  |  |  |  |  |  |  |  | 0 0.0 | 3 42.9 | 4 100.0 |  |  |  |  | 2 |  |
|  | | | | | | | | | | | | | | | | | | |
| *Aspergillus versicolor* species complex | | | | | | | | | | | | | | | | | |  |
| Manogepix (1) |  |  |  | 0 0.0 | 1 100.0 |  |  |  |  |  |  |  |  |  |  |  |  |  |
| Voriconazole (1) |  |  |  |  |  |  |  |  | 0 0.0 | 1 100.0 |  |  |  |  |  |  |  |  |
| Anidulafungin (1) |  |  | 0 0.0 | 1 100.0 |  |  |  |  |  |  |  |  |  |  |  |  |  |  |
| Micafungin (1) |  |  | 0 0.0 | 1 100.0 |  |  |  |  |  |  |  |  |  |  |  |  |  |  |
| Itraconazole (1) |  |  |  |  |  |  |  |  |  | 0 0.0 | 1 100.0 |  |  |  |  |  |  |  |
| Amphotericin B (1) |  |  |  |  |  |  |  |  |  |  | 0 0.0 | 1 100.0 |  |  |  |  |  |  |
|  | | | | | | | | | | | | | | | | | | |
| *Aspergillus welwitschiae* | | | | | | | | | | | | | | | | | |  |
| Manogepix (1) |  |  |  | 0 0.0 | 1 100.0 |  |  |  |  |  |  |  |  |  |  |  |  |  |
| Voriconazole (1) |  |  |  |  |  |  |  |  |  | 0 0.0 | 1 100.0 |  |  |  |  |  |  |  |
| Anidulafungin (1) |  | 0 0.0 | 1 100.0 |  |  |  |  |  |  |  |  |  |  |  |  |  |  |  |
| Micafungin (1) |  |  | 0 0.0 | 1 100.0 |  |  |  |  |  |  |  |  |  |  |  |  |  |  |
| Itraconazole (1) |  |  |  |  |  |  |  |  |  | 0 0.0 | 1 100.0 |  |  |  |  |  |  |  |
| Amphotericin B (1) |  |  |  |  |  |  | 0 0.0 | 1 100.0 |  |  |  |  |  |  |  |  |  |  |
|  | | | | | | | | | | | | | | | | | | |
| *Aureobasidium pullulans* | | | | | | | | | | | | | | | | | |  |
| Manogepix (2) |  |  | 0 0.0 | 2 100.0 |  |  |  |  |  |  |  |  |  |  |  |  | 0.008 |  |
| Voriconazole (2) |  |  |  |  | 0 0.0 | 2 100.0 |  |  |  |  |  |  |  |  |  |  | 0.03 |  |
| Anidulafungin (2) |  |  |  |  |  |  |  | 0 0.0 | 1 50.0 | 0 50.0 | 1 100.0 |  |  |  |  |  | 0.25 |  |
| Micafungin (2) |  |  |  |  |  |  |  | 0 0.0 | 1 50.0 | 0 50.0 | 0 50.0 | 1 100.0 |  |  |  |  | 0.25 |  |
| Itraconazole (2) |  |  |  |  |  |  | 0 0.0 | 1 50.0 | 1 100.0 |  |  |  |  |  |  |  | 0.12 |  |
| Amphotericin B (2) |  |  |  |  |  |  |  | 0 0.0 | 1 50.0 | 1 100.0 |  |  |  |  |  |  | 0.25 |  |
|  | | | | | | | | | | | | | | | | | | |
| *Coprinopsis cinerea* | | | | | | | | | | | | | | | | | |  |
| Manogepix (1) |  | 0 0.0 | 1 100.0 |  |  |  |  |  |  |  |  |  |  |  |  |  |  |  |
| Voriconazole (1) |  |  |  |  |  |  |  | 0 0.0 | 1 100.0 |  |  |  |  |  |  |  |  |  |
| Anidulafungin (1) |  |  |  |  |  |  |  |  |  |  |  | 0 0.0 | 1 100.0 |  |  |  |  |  |
| Micafungin (1) |  |  |  |  |  |  |  |  |  |  |  |  | 0 0.0 |  | 1 100.0 |  |  |  |
| Itraconazole (1) |  |  |  |  |  |  |  |  |  | 0 0.0 | 1 100.0 |  |  |  |  |  |  |  |
| Amphotericin B (1) |  |  |  |  |  |  |  |  |  | 0 0.0 | 1 100.0 |  |  |  |  |  |  |  |
|  | | | | | | | | | | | | | | | | | | |
| *Exophiala attenuata* | | | | | | | | | | | | | | | | | |  |
| Manogepix (2) |  |  | 0 0.0 | 1 50.0 | 1 100.0 |  |  |  |  |  |  |  |  |  |  |  | 0.008 |  |
| Voriconazole (2) |  |  |  |  | 0 0.0 | 1 50.0 | 0 50.0 | 1 100.0 |  |  |  |  |  |  |  |  | 0.03 |  |
| Anidulafungin (2) |  |  |  |  |  |  |  |  | 0 0.0 | 1 50.0 | 0 50.0 | 1 100.0 |  |  |  |  | 0.5 |  |
| Micafungin (2) |  |  |  |  |  |  | 0 0.0 | 2 100.0 |  |  |  |  |  |  |  |  | 0.12 |  |
| Itraconazole (2) |  |  |  |  |  |  |  | 0 0.0 | 2 100.0 |  |  |  |  |  |  |  | 0.25 |  |
| Amphotericin B (2) |  |  |  |  |  |  |  |  |  |  | 0 0.0 | 2 100.0 |  |  |  |  | 2 |  |
|  | | | | | | | | | | | | | | | | | | |
| *Fusarium annulatum* | | | | | | | | | | | | | | | | | |  |
| Manogepix (2) |  |  | 0 0.0 | 1 50.0 | 0 50.0 | 1 100.0 |  |  |  |  |  |  |  |  |  |  | 0.008 |  |
| Voriconazole (2) |  |  |  |  |  |  |  |  |  |  |  |  | 0 0.0 | 1 50.0 | 1 100.0 |  | 8 |  |
| Anidulafungin (2) |  |  |  |  |  |  |  |  |  |  |  |  | 0 0.0 |  | 2 100.0 |  | >4 |  |
| Micafungin (2) |  |  |  |  |  |  |  |  |  |  |  |  | 0 0.0 |  | 2 100.0 |  | >4 |  |
| Itraconazole (2) |  |  |  |  |  |  |  |  |  |  |  |  |  | 0 0.0 | 2 100.0 |  | >8 |  |
| Amphotericin B (2) |  |  |  |  |  |  |  |  |  |  | 0 0.0 | 2 100.0 |  |  |  |  | 2 |  |
|  | | | | | | | | | | | | | | | | | | |
| *Fusarium dimerum* species complex | | | | | | | | | | | | | | | | | |  |
| Manogepix (1) |  |  |  |  |  | 0 0.0 | 1 100.0 |  |  |  |  |  |  |  |  |  |  |  |
| Voriconazole (1) |  |  |  |  |  |  |  |  |  |  |  |  | 0 0.0 | 1 100.0 |  |  |  |  |
| Anidulafungin (1) |  |  |  |  |  |  |  |  |  |  |  |  | 0 0.0 |  | 1 100.0 |  |  |  |
| Micafungin (1) |  |  |  |  |  |  |  |  |  |  | 0 0.0 | 1 100.0 |  |  |  |  |  |  |
| Itraconazole (1) |  |  |  |  |  |  |  |  |  |  |  |  |  | 0 0.0 | 1 100.0 |  |  |  |
| Amphotericin B (1) |  |  |  |  |  |  |  |  |  |  | 0 0.0 | 1 100.0 |  |  |  |  |  |  |
|  | | | | | | | | | | | | | | | | | | |
| *Fusarium falciforme* | | | | | | | | | | | | | | | | | |  |
| Manogepix (1) |  |  |  | 0 0.0 | 1 100.0 |  |  |  |  |  |  |  |  |  |  |  |  |  |
| Voriconazole (1) |  |  |  |  |  |  |  |  |  |  |  |  |  | 0 0.0 | 1 100.0 |  |  |  |
| Anidulafungin (1) |  |  |  |  |  |  |  |  |  |  |  |  | 0 0.0 |  | 1 100.0 |  |  |  |
| Micafungin (1) |  |  |  |  |  |  |  |  |  |  |  |  | 0 0.0 |  | 1 100.0 |  |  |  |
| Itraconazole (1) |  |  |  |  |  |  |  |  |  |  |  |  |  | 0 0.0 | 1 100.0 |  |  |  |
| Amphotericin B (1) |  |  |  |  |  |  |  |  |  | 0 0.0 | 1 100.0 |  |  |  |  |  |  |  |
|  | | | | | | | | | | | | | | | | | | |
| *Fusarium incarnatum-equiseti* species complex | | | | | | | | | | | | | | | | | |  |
| Manogepix (4) |  | 1 25.0 | 0 25.0 | 0 25.0 | 0 25.0 | 0 25.0 | 0 25.0 | 1 50.0 | 0 50.0 | 0 50.0 | 0 50.0 | 1 75.0 | 0 75.0 | 1 100.0 |  |  | 0.12 |  |
| Voriconazole (4) |  |  |  |  |  |  |  |  |  |  | 0 0.0 | 3 75.0 | 1 100.0 |  |  |  | 2 |  |
| Anidulafungin (4) |  |  |  |  |  |  |  |  |  |  |  | 0 0.0 | 1 25.0 | 0 25.0 | 3 100.0 |  | >8 |  |
| Micafungin (4) |  |  |  |  |  |  |  |  |  |  | 0 0.0 | 1 25.0 | 0 25.0 | 0 25.0 | 3 100.0 |  | >8 |  |
| Itraconazole (4) |  |  |  |  |  |  |  |  |  |  | 0 0.0 | 1 25.0 | 0 25.0 | 0 25.0 | 3 100.0 |  | >8 |  |
| Amphotericin B (4) |  |  |  |  |  |  |  |  |  | 0 0.0 | 4 100.0 |  |  |  |  |  | 1 |  |
|  | | | | | | | | | | | | | | | | | | |
| *Fusarium oxysporum* species complex | | | | | | | | | | | | | | | | | |  |
| Manogepix (8) |  |  | 0 0.0 | 1 12.5 | 2 37.5 | 1 50.0 | 3 87.5 | 0 87.5 | 0 87.5 | 0 87.5 | 0 87.5 | 0 87.5 | 1 100.0 |  |  |  | 0.03 |  |
| Voriconazole (8) |  |  |  |  |  |  |  |  |  |  | 0 0.0 | 1 12.5 | 3 50.0 | 3 87.5 | 1 100.0 |  | 4 |  |
| Anidulafungin (8) |  |  |  |  |  |  |  |  |  |  |  |  | 0 0.0 |  | 8 100.0 |  | >4 |  |
| Micafungin (8) |  |  |  |  |  |  |  |  |  |  |  |  | 0 0.0 |  | 8 100.0 |  | >4 |  |
| Itraconazole (8) |  |  |  |  |  |  |  |  |  |  |  |  |  | 0 0.0 | 8 100.0 |  | >8 |  |
| Amphotericin B (8) |  |  |  |  |  |  |  |  |  | 0 0.0 | 2 25.0 | 5 87.5 | 1 100.0 |  |  |  | 2 |  |
|  | | | | | | | | | | | | | | | | | | |
| *Fusarium petroliphilum* | | | | | | | | | | | | | | | | | |  |
| Manogepix (1) |  |  |  | 0 0.0 | 1 100.0 |  |  |  |  |  |  |  |  |  |  |  |  |  |
| Voriconazole (1) |  |  |  |  |  |  |  |  |  |  |  |  |  | 0 0.0 | 1 100.0 |  |  |  |
| Anidulafungin (1) |  |  |  |  |  |  |  |  |  |  |  |  | 0 0.0 |  | 1 100.0 |  |  |  |
| Micafungin (1) |  |  |  |  |  |  |  |  |  |  |  |  | 0 0.0 |  | 1 100.0 |  |  |  |
| Itraconazole (1) |  |  |  |  |  |  |  |  |  |  |  |  |  | 0 0.0 | 1 100.0 |  |  |  |
| Amphotericin B (1) |  |  |  |  |  |  |  |  | 0 0.0 | 1 100.0 |  |  |  |  |  |  |  |  |
|  | | | | | | | | | | | | | | | | | | |
| *Fusarium solani* | | | | | | | | | | | | | | | | | |  |
| Manogepix (2) |  | 0 0.0 | 1 50.0 | 0 50.0 | 1 100.0 |  |  |  |  |  |  |  |  |  |  |  | 0.004 |  |
| Voriconazole (2) |  |  |  |  |  |  |  |  |  |  |  |  | 0 0.0 | 1 50.0 | 1 100.0 |  | 8 |  |
| Anidulafungin (2) |  | 0 0.0 | 1 50.0 | 0 50.0 | 0 50.0 | 0 50.0 | 0 50.0 | 0 50.0 | 0 50.0 | 0 50.0 | 0 50.0 | 0 50.0 | 0 50.0 |  | 1 100.0 |  | 0.004 |  |
| Micafungin (2) |  |  | 0 0.0 | 1 50.0 | 0 50.0 | 0 50.0 | 0 50.0 | 0 50.0 | 0 50.0 | 0 50.0 | 0 50.0 | 0 50.0 | 0 50.0 |  | 1 100.0 |  | 0.008 |  |
| Itraconazole (2) |  |  |  |  |  |  |  |  |  |  |  |  |  | 0 0.0 | 2 100.0 |  | >8 |  |
| Amphotericin B (2) |  |  |  |  |  |  |  |  |  |  | 0 0.0 | 1 50.0 | 1 100.0 |  |  |  | 2 |  |
|  | | | | | | | | | | | | | | | | | | |
| *Lichtheimia corymbifera* | | | | | | | | | | | | | | | | | |  |
| Manogepix (8) |  |  |  |  |  |  |  |  |  |  |  | 0 0.0 | 2 25.0 |  | 6 100.0 |  | >4 |  |
| Voriconazole (8) |  |  |  |  |  |  |  |  |  |  |  |  |  | 0 0.0 | 8 100.0 |  | >8 |  |
| Anidulafungin (8) |  |  |  |  |  |  |  |  |  |  |  | 0 0.0 | 2 25.0 |  | 6 100.0 |  | >4 |  |
| Micafungin (8) |  |  |  |  |  |  |  |  |  |  |  |  | 0 0.0 |  | 8 100.0 |  | >4 |  |
| Itraconazole (8) |  |  |  |  |  |  |  |  |  | 0 0.0 | 7 87.5 | 1 100.0 |  |  |  |  | 1 |  |
| Amphotericin B (8) |  |  |  |  |  | 0 0.0 | 1 12.5 | 0 12.5 | 0 12.5 | 6 87.5 | 1 100.0 |  |  |  |  |  | 0.5 |  |
|  | | | | | | | | | | | | | | | | | | |
| *Lichtheimia ramosa* | | | | | | | | | | | | | | | | | |  |
| Manogepix (1) |  |  |  |  |  |  |  |  |  |  |  |  | 0 0.0 |  | 1 100.0 |  |  |  |
| Voriconazole (1) |  |  |  |  |  |  |  |  |  |  |  |  |  | 0 0.0 | 1 100.0 |  |  |  |
| Anidulafungin (1) |  |  |  |  |  |  |  |  |  |  |  | 0 0.0 | 1 100.0 |  |  |  |  |  |
| Micafungin (1) |  |  |  |  |  |  |  |  |  |  |  |  | 0 0.0 |  | 1 100.0 |  |  |  |
| Itraconazole (1) |  |  |  |  |  |  |  |  |  |  |  | 0 0.0 | 1 100.0 |  |  |  |  |  |
| Amphotericin B (1) |  |  |  |  |  | 0 0.0 | 1 100.0 |  |  |  |  |  |  |  |  |  |  |  |
|  | | | | | | | | | | | | | | | | | | |
| *Medicopsis romeroi* | | | | | | | | | | | | | | | | | |  |
| Manogepix (2) |  |  |  |  | 0 0.0 | 1 50.0 | 0 50.0 | 1 100.0 |  |  |  |  |  |  |  |  | 0.03 |  |
| Voriconazole (2) |  |  |  |  |  |  |  | 0 0.0 | 1 50.0 | 0 50.0 | 0 50.0 | 0 50.0 | 0 50.0 | 0 50.0 | 1 100.0 |  | 0.25 |  |
| Anidulafungin (2) |  |  |  | 0 0.0 | 1 50.0 | 0 50.0 | 0 50.0 | 0 50.0 | 0 50.0 | 0 50.0 | 0 50.0 | 0 50.0 | 0 50.0 |  | 1 100.0 |  | 0.015 |  |
| Micafungin (2) |  |  |  |  |  | 0 0.0 | 1 50.0 | 0 50.0 | 0 50.0 | 0 50.0 | 0 50.0 | 0 50.0 | 0 50.0 |  | 1 100.0 |  | 0.06 |  |
| Itraconazole (2) |  |  |  |  |  |  |  |  |  | 0 0.0 | 1 50.0 | 0 50.0 | 0 50.0 | 0 50.0 | 1 100.0 |  | 1 |  |
| Amphotericin B (2) |  |  |  |  |  |  |  |  | 0 0.0 | 1 50.0 | 1 100.0 |  |  |  |  |  | 0.5 |  |
|  | | | | | | | | | | | | | | | | | | |
| *Microascus cirrosus* | | | | | | | | | | | | | | | | | |  |
| Manogepix (1) |  |  | 0 0.0 | 1 100.0 |  |  |  |  |  |  |  |  |  |  |  |  |  |  |
| Voriconazole (1) |  |  |  |  |  |  |  |  |  |  |  |  |  | 0 0.0 | 1 100.0 |  |  |  |
| Anidulafungin (1) |  |  |  |  |  |  |  |  |  |  |  |  | 0 0.0 |  | 1 100.0 |  |  |  |
| Micafungin (1) |  |  |  |  |  |  |  |  |  |  |  |  | 0 0.0 |  | 1 100.0 |  |  |  |
| Itraconazole (1) |  |  |  |  |  |  |  |  |  |  |  |  |  | 0 0.0 | 1 100.0 |  |  |  |
| Amphotericin B (1) |  |  |  |  |  |  |  |  |  |  |  |  | 0 0.0 |  | 1 100.0 |  |  |  |
|  | | | | | | | | | | | | | | | | | | |
| *Monascus ruber* | | | | | | | | | | | | | | | | | |  |
| Manogepix (1) |  |  |  |  | 0 0.0 | 1 100.0 |  |  |  |  |  |  |  |  |  |  |  |  |
| Voriconazole (1) |  |  |  |  |  |  |  |  |  |  |  |  |  | 0 0.0 | 1 100.0 |  |  |  |
| Anidulafungin (1) |  |  |  |  |  | 0 0.0 | 1 100.0 |  |  |  |  |  |  |  |  |  |  |  |
| Micafungin (1) |  |  |  | 0 0.0 | 1 100.0 |  |  |  |  |  |  |  |  |  |  |  |  |  |
| Itraconazole (1) |  |  |  |  |  |  |  |  |  |  |  |  | 0 0.0 | 1 100.0 |  |  |  |  |
| Amphotericin B (1) |  |  |  |  |  |  | 0 0.0 | 1 100.0 |  |  |  |  |  |  |  |  |  |  |
|  | | | | | | | | | | | | | | | | | | |
| *Mucor circinelloides* | | | | | | | | | | | | | | | | | |  |
| Manogepix (8) |  |  |  |  |  |  |  | 0 0.0 | 2 25.0 | 0 25.0 | 1 37.5 | 1 50.0 | 1 62.5 |  | 3 100.0 |  | 2 |  |
| Voriconazole (8) |  |  |  |  |  |  |  |  |  |  |  |  |  | 0 0.0 | 8 100.0 |  | >8 |  |
| Anidulafungin (8) |  |  |  |  |  |  |  |  |  |  |  | 0 0.0 | 1 12.5 |  | 7 100.0 |  | >4 |  |
| Micafungin (8) |  |  |  |  |  |  |  |  |  |  |  |  | 0 0.0 |  | 8 100.0 |  | >4 |  |
| Itraconazole (8) |  |  |  |  |  |  |  |  |  | 0 0.0 | 1 12.5 | 2 37.5 | 0 37.5 | 2 62.5 | 3 100.0 |  | 8 |  |
| Amphotericin B (8) |  |  |  |  |  | 0 0.0 | 2 25.0 | 0 25.0 | 3 62.5 | 3 100.0 |  |  |  |  |  |  | 0.25 |  |
|  | | | | | | | | | | | | | | | | | | |
| *Mucor circinelloides/ Mucor ramosissimus* | | | | | | | | | | | | | | | | | |  |
| Manogepix (2) |  |  |  |  |  |  |  |  |  | 0 0.0 | 1 50.0 | 1 100.0 |  |  |  |  | 1 |  |
| Voriconazole (2) |  |  |  |  |  |  |  |  |  |  |  |  |  | 0 0.0 | 2 100.0 |  | >8 |  |
| Anidulafungin (2) |  |  |  |  |  |  |  |  |  |  |  | 0 0.0 | 1 50.0 |  | 1 100.0 |  | 4 |  |
| Micafungin (2) |  |  |  |  |  |  |  |  |  |  |  |  | 0 0.0 |  | 2 100.0 |  | >4 |  |
| Itraconazole (2) |  |  |  |  |  |  |  |  |  |  | 0 0.0 | 1 50.0 | 1 100.0 |  |  |  | 2 |  |
| Amphotericin B (2) |  |  |  |  |  |  |  | 0 0.0 | 2 100.0 |  |  |  |  |  |  |  | 0.25 |  |
|  | | | | | | | | | | | | | | | | | | |
| *Mucor indicus* | | | | | | | | | | | | | | | | | |  |
| Manogepix (1) |  |  |  |  |  |  |  |  |  | 0 0.0 | 1 100.0 |  |  |  |  |  |  |  |
| Voriconazole (1) |  |  |  |  |  |  |  |  |  |  |  |  |  | 0 0.0 | 1 100.0 |  |  |  |
| Anidulafungin (1) |  |  |  |  |  |  |  |  |  |  |  |  | 0 0.0 |  | 1 100.0 |  |  |  |
| Micafungin (1) |  |  |  |  |  |  |  |  |  |  |  |  | 0 0.0 |  | 1 100.0 |  |  |  |
| Itraconazole (1) |  |  |  |  |  |  |  |  |  |  |  |  | 0 0.0 | 1 100.0 |  |  |  |  |
| Amphotericin B (1) |  |  |  |  |  |  |  |  | 0 0.0 | 1 100.0 |  |  |  |  |  |  |  |  |
|  | | | | | | | | | | | | | | | | | | |
| *Paecilomyces lilacinus* | | | | | | | | | | | | | | | | | |  |
| Manogepix (15) |  |  |  | 11 73.3 | 4 100.0 |  |  |  |  |  |  |  |  |  |  |  | ≤0.008 | 0.015 |
| Voriconazole (15) |  |  |  |  |  | 0 0.0 | 1 6.7 | 0 6.7 | 9 66.7 | 4 93.3 | 1 100.0 |  |  |  |  |  | 0.25 | 0.5 |
| Anidulafungin (15) |  |  |  | 0 0.0 | 1 6.7 | 1 13.3 | 4 40.0 | 2 53.3 | 4 80.0 | 2 93.3 | 0 93.3 | 0 93.3 | 0 93.3 |  | 1 100.0 |  | 0.12 | 0.5 |
| Micafungin (15) |  |  |  | 0 0.0 | 4 26.7 | 4 53.3 | 4 80.0 | 2 93.3 | 0 93.3 | 0 93.3 | 0 93.3 | 0 93.3 | 0 93.3 |  | 1 100.0 |  | 0.03 | 0.12 |
| Itraconazole (15) |  |  |  |  |  |  |  |  | 0 0.0 | 1 6.7 | 0 6.7 | 3 26.7 | 2 40.0 | 0 40.0 | 9 100.0 |  | >8 | >8 |
| Amphotericin B (15) |  |  |  |  |  |  |  |  |  |  | 0 0.0 | 1 6.7 |  |  | 14 100.0 |  | >2 | >2 |
|  | | | | | | | | | | | | | | | | | | |
| *Penicillium citrinum* | | | | | | | | | | | | | | | | | |  |
| Manogepix (1) |  |  | 0 0.0 | 1 100.0 |  |  |  |  |  |  |  |  |  |  |  |  |  |  |
| Voriconazole (1) |  |  |  |  |  |  |  |  |  |  |  |  |  | 0 0.0 | 1 100.0 |  |  |  |
| Anidulafungin (1) |  |  |  | 0 0.0 | 1 100.0 |  |  |  |  |  |  |  |  |  |  |  |  |  |
| Micafungin (1) |  | 0 0.0 | 1 100.0 |  |  |  |  |  |  |  |  |  |  |  |  |  |  |  |
| Itraconazole (1) |  |  |  |  |  |  |  |  |  |  | 0 0.0 | 1 100.0 |  |  |  |  |  |  |
| Amphotericin B (1) |  |  |  |  |  |  |  |  |  | 0 0.0 | 1 100.0 |  |  |  |  |  |  |  |
|  | | | | | | | | | | | | | | | | | | |
| *Penicillium onobense* | | | | | | | | | | | | | | | | | |  |
| Manogepix (1) |  |  | 0 0.0 | 1 100.0 |  |  |  |  |  |  |  |  |  |  |  |  |  |  |
| Voriconazole (1) |  |  |  |  |  |  |  |  | 0 0.0 | 1 100.0 |  |  |  |  |  |  |  |  |
| Anidulafungin (1) |  |  | 0 0.0 | 1 100.0 |  |  |  |  |  |  |  |  |  |  |  |  |  |  |
| Micafungin (1) |  |  |  | 0 0.0 | 1 100.0 |  |  |  |  |  |  |  |  |  |  |  |  |  |
| Itraconazole (1) |  |  |  |  |  |  |  |  | 0 0.0 | 1 100.0 |  |  |  |  |  |  |  |  |
| Amphotericin B (1) |  |  |  |  |  |  |  |  |  | 0 0.0 | 1 100.0 |  |  |  |  |  |  |  |
|  | | | | | | | | | | | | | | | | | | |
| *Phaeoacremonium parasiticum* | | | | | | | | | | | | | | | | | |  |
| Manogepix (1) |  |  |  |  |  | 0 0.0 | 1 100.0 |  |  |  |  |  |  |  |  |  |  |  |
| Voriconazole (1) |  |  |  |  |  |  |  | 0 0.0 | 1 100.0 |  |  |  |  |  |  |  |  |  |
| Anidulafungin (1) |  |  |  |  |  |  |  |  |  |  | 0 0.0 | 1 100.0 |  |  |  |  |  |  |
| Micafungin (1) |  |  |  |  |  |  |  | 0 0.0 | 1 100.0 |  |  |  |  |  |  |  |  |  |
| Itraconazole (1) |  |  |  |  |  |  |  |  |  |  |  | 0 0.0 | 1 100.0 |  |  |  |  |  |
| Amphotericin B (1) |  |  |  |  |  |  |  |  | 0 0.0 | 1 100.0 |  |  |  |  |  |  |  |  |
|  | | | | | | | | | | | | | | | | | | |
| *Pleurostomophora richardsiae* | | | | | | | | | | | | | | | | | |  |
| Manogepix (1) |  |  |  |  |  | 0 0.0 | 1 100.0 |  |  |  |  |  |  |  |  |  |  |  |
| Voriconazole (1) |  |  |  |  |  |  |  |  | 0 0.0 | 1 100.0 |  |  |  |  |  |  |  |  |
| Anidulafungin (1) |  |  |  |  |  |  |  |  |  |  |  |  | 0 0.0 |  | 1 100.0 |  |  |  |
| Micafungin (1) |  |  |  |  |  |  |  |  |  |  |  |  | 0 0.0 |  | 1 100.0 |  |  |  |
| Itraconazole (1) |  |  |  |  |  |  |  |  |  | 0 0.0 | 1 100.0 |  |  |  |  |  |  |  |
| Amphotericin B (1) |  |  |  |  |  |  |  |  |  | 0 0.0 | 1 100.0 |  |  |  |  |  |  |  |
|  | | | | | | | | | | | | | | | | | | |
| *Pseudopithomyces sacchari*  (*Pithmyces sacchari*) | | | | | | | | | | | | | | | | | |  |
| Manogepix (1) |  |  |  |  |  |  |  | 0 0.0 | 1 100.0 |  |  |  |  |  |  |  |  |  |
| Voriconazole (1) |  |  |  |  |  |  | 0 0.0 | 1 100.0 |  |  |  |  |  |  |  |  |  |  |
| Anidulafungin (1) |  |  | 0 0.0 | 1 100.0 |  |  |  |  |  |  |  |  |  |  |  |  |  |  |
| Micafungin (1) |  |  | 0 0.0 | 1 100.0 |  |  |  |  |  |  |  |  |  |  |  |  |  |  |
| Itraconazole (1) |  |  |  |  |  | 0 0.0 | 1 100.0 |  |  |  |  |  |  |  |  |  |  |  |
| Amphotericin B (1) |  |  |  |  |  | 1 100.0 |  |  |  |  |  |  |  |  |  |  |  |  |
|  | | | | | | | | | | | | | | | | | | |
| *Rasamsonia argillacea* | | | | | | | | | | | | | | | | | |  |
| Manogepix (6) |  | 0 0.0 | 2 33.3 | 2 66.7 | 2 100.0 |  |  |  |  |  |  |  |  |  |  |  | 0.008 |  |
| Voriconazole (6) |  |  |  |  |  |  |  |  |  |  |  |  | 0 0.0 | 1 16.7 | 5 100.0 |  | >8 |  |
| Anidulafungin (6) |  | 0 0.0 | 2 33.3 | 2 66.7 | 2 100.0 |  |  |  |  |  |  |  |  |  |  |  | 0.008 |  |
| Micafungin (6) |  | 0 0.0 | 3 50.0 | 3 100.0 |  |  |  |  |  |  |  |  |  |  |  |  | 0.004 |  |
| Itraconazole (6) |  |  |  |  |  |  |  |  | 0 0.0 | 1 16.7 | 0 16.7 | 2 50.0 | 0 50.0 | 0 50.0 | 3 100.0 |  | 2 |  |
| Amphotericin B (6) |  |  |  |  |  |  |  |  |  | 0 0.0 | 3 50.0 | 2 83.3 | 1 100.0 |  |  |  | 1 |  |
|  | | | | | | | | | | | | | | | | | | |
| *Rasamsonia argillacea* species complex | | | | | | | | | | | | | | | | | |  |
| Manogepix (6) |  |  |  | 4 66.7 | 2 100.0 |  |  |  |  |  |  |  |  |  |  |  | ≤0.008 |  |
| Voriconazole (6) |  |  |  |  |  |  |  |  |  |  |  |  | 0 0.0 | 1 16.7 | 5 100.0 |  | >8 |  |
| Anidulafungin (6) |  |  |  | 2 33.3 | 3 83.3 | 1 100.0 |  |  |  |  |  |  |  |  |  |  | 0.015 |  |
| Micafungin (6) |  |  |  | 5 83.3 | 1 100.0 |  |  |  |  |  |  |  |  |  |  |  | ≤0.008 |  |
| Itraconazole (6) |  |  |  |  |  |  |  |  | 0 0.0 | 1 16.7 | 2 50.0 | 0 50.0 | 0 50.0 | 1 66.7 | 2 100.0 |  | 1 |  |
| Amphotericin B (6) |  |  |  |  |  |  |  | 0 0.0 | 1 16.7 | 1 33.3 | 3 83.3 | 1 100.0 |  |  |  |  | 1 |  |
|  | | | | | | | | | | | | | | | | | | |
| *Rhizomucor pusillus* | | | | | | | | | | | | | | | | | |  |
| Manogepix (6) |  |  |  |  |  |  |  |  |  | 0 0.0 | 1 16.7 | 0 16.7 | 2 50.0 |  | 3 100.0 |  | 4 |  |
| Voriconazole (6) |  |  |  |  |  |  |  |  |  |  |  |  |  | 0 0.0 | 6 100.0 |  | >8 |  |
| Anidulafungin (6) |  |  |  |  |  |  |  |  |  |  |  | 0 0.0 | 3 50.0 |  | 3 100.0 |  | 4 |  |
| Micafungin (6) |  |  |  |  |  |  |  |  |  |  |  |  | 0 0.0 |  | 6 100.0 |  | >4 |  |
| Itraconazole (6) |  |  |  |  |  |  |  |  | 0 0.0 | 2 33.3 | 2 66.7 | 1 83.3 | 1 100.0 |  |  |  | 1 |  |
| Amphotericin B (6) |  |  |  |  |  | 0 0.0 | 1 16.7 | 0 16.7 | 1 33.3 | 3 83.3 | 1 100.0 |  |  |  |  |  | 0.5 |  |
|  | | | | | | | | | | | | | | | | | | |
| *Rhizopus oryzae* species complex | | | | | | | | | | | | | | | | | |  |
| Manogepix (7) |  |  |  |  |  |  |  |  | 0 0.0 | 1 14.3 | 0 14.3 | 0 14.3 | 1 28.6 |  | 5 100.0 |  | >4 |  |
| Voriconazole (7) |  |  |  |  |  |  |  |  |  |  |  |  | 0 0.0 | 3 42.9 | 4 100.0 |  | >8 |  |
| Anidulafungin (7) |  |  |  |  |  |  |  |  |  |  |  |  | 0 0.0 |  | 7 100.0 |  | >4 |  |
| Micafungin (7) |  |  |  |  |  |  |  |  |  |  |  |  | 0 0.0 |  | 7 100.0 |  | >4 |  |
| Itraconazole (7) |  |  |  |  |  |  |  |  |  | 0 0.0 | 4 57.1 | 1 71.4 | 1 85.7 | 1 100.0 |  |  | 1 |  |
| Amphotericin B (7) |  |  |  |  |  |  | 0 0.0 | 4 57.1 | 1 71.4 | 1 85.7 | 1 100.0 |  |  |  |  |  | 0.12 |  |
|  | | | | | | | | | | | | | | | | | | |
| *Sarocladium kiliense* | | | | | | | | | | | | | | | | | |  |
| Manogepix (4) |  |  |  | 0 0.0 | 1 25.0 | 1 50.0 | 1 75.0 | 1 100.0 |  |  |  |  |  |  |  |  | 0.03 |  |
| Voriconazole (4) |  |  |  |  |  |  |  |  |  |  | 0 0.0 | 3 75.0 | 1 100.0 |  |  |  | 2 |  |
| Anidulafungin (4) |  |  |  |  |  |  |  |  |  |  |  | 0 0.0 | 1 25.0 |  | 3 100.0 |  | >4 |  |
| Micafungin (4) |  |  |  |  |  | 0 0.0 | 1 25.0 | 0 25.0 | 0 25.0 | 0 25.0 | 0 25.0 | 0 25.0 | 1 50.0 |  | 2 100.0 |  | 4 |  |
| Itraconazole (4) |  |  |  |  |  |  |  |  |  |  |  |  |  | 0 0.0 | 4 100.0 |  | >8 |  |
| Amphotericin B (4) |  |  |  |  |  |  |  |  |  |  | 0 0.0 | 1 25.0 | 1 50.0 |  | 2 100.0 |  | 4 |  |
|  | | | | | | | | | | | | | | | | | | |
| *Scedosporium apiospermum* | | | | | | | | | | | | | | | | | |  |
| Manogepix (9) |  | 0 0.0 | 1 11.1 | 1 22.2 | 4 66.7 | 2 88.9 | 1 100.0 |  |  |  |  |  |  |  |  |  | 0.015 |  |
| Voriconazole (9) |  |  |  |  |  |  |  |  | 0 0.0 | 1 11.1 | 7 88.9 | 1 100.0 |  |  |  |  | 1 |  |
| Anidulafungin (9) |  |  |  |  |  |  |  |  |  | 0 0.0 | 1 11.1 | 6 77.8 | 2 100.0 |  |  |  | 2 |  |
| Micafungin (9) |  |  |  |  |  |  | 0 0.0 | 4 44.4 | 1 55.6 | 3 88.9 | 0 88.9 | 0 88.9 | 0 88.9 | 0 88.9 | 1 100.0 |  | 0.25 |  |
| Itraconazole (9) |  |  |  |  |  |  |  |  |  |  | 0 0.0 | 1 11.1 | 0 11.1 | 1 22.2 | 7 100.0 |  | >8 |  |
| Amphotericin B (9) |  |  |  |  |  |  |  | 0 0.0 | 2 22.2 | 0 22.2 | 3 55.6 | 3 88.9 | 0 88.9 |  | 1 100.0 |  | 1 |  |
|  | | | | | | | | | | | | | | | | | | |
| *Scedosporium dehoogii* | | | | | | | | | | | | | | | | | |  |
| Manogepix (3) |  |  |  |  | 0 0.0 | 2 66.7 | 1 100.0 |  |  |  |  |  |  |  |  |  | 0.03 |  |
| Voriconazole (3) |  |  |  |  |  |  |  |  | 0 0.0 | 1 33.3 | 1 66.7 | 1 100.0 |  |  |  |  | 1 |  |
| Anidulafungin (3) |  |  |  |  |  |  |  |  |  |  | 0 0.0 | 1 33.3 | 0 33.3 | 2 100.0 |  |  | 8 |  |
| Micafungin (3) |  |  |  |  |  |  |  | 0 0.0 | 1 33.3 | 0 33.3 | 0 33.3 | 0 33.3 | 0 33.3 | 0 33.3 | 2 100.0 |  | >8 |  |
| Itraconazole (3) |  |  |  |  |  |  |  |  |  |  |  | 0 0.0 | 1 33.3 | 1 66.7 | 1 100.0 |  | 8 |  |
| Amphotericin B (3) |  |  |  |  |  |  |  |  |  |  |  | 0 0.0 |  |  | 3 100.0 |  | >2 |  |
|  | | | | | | | | | | | | | | | | | | |
| *Scedosporium minutisporum* | | | | | | | | | | | | | | | | | |  |
| Manogepix (2) |  |  | 0 0.0 | 1 50.0 | 1 100.0 |  |  |  |  |  |  |  |  |  |  |  | 0.008 |  |
| Voriconazole (2) |  |  |  |  |  |  |  | 0 0.0 | 2 100.0 |  |  |  |  |  |  |  | 0.25 |  |
| Anidulafungin (2) |  |  |  |  |  |  |  |  |  | 0 0.0 | 1 50.0 | 1 100.0 |  |  |  |  | 1 |  |
| Micafungin (2) |  |  |  |  |  |  |  | 0 0.0 | 1 50.0 | 1 100.0 |  |  |  |  |  |  | 0.25 |  |
| Itraconazole (2) |  |  |  |  |  |  |  |  |  |  | 0 0.0 | 2 100.0 |  |  |  |  | 2 |  |
| Amphotericin B (2) |  |  |  |  |  |  |  |  |  |  |  | 0 0.0 | 2 100.0 |  |  |  | 4 |  |
|  | | | | | | | | | | | | | | | | | | |
| *Scopulariopsis brevicaulis* | | | | | | | | | | | | | | | | | |  |
| Manogepix (2) |  | 1 50.0 | 0 50.0 | 1 100.0 |  |  |  |  |  |  |  |  |  |  |  |  | ≤0.002 |  |
| Voriconazole (2) |  |  |  |  |  |  |  |  |  | 0 0.0 | 1 50.0 | 0 50.0 | 0 50.0 | 0 50.0 | 1 100.0 |  | 1 |  |
| Anidulafungin (2) |  |  |  |  |  |  |  |  | 0 0.0 | 1 50.0 | 1 100.0 |  |  |  |  |  | 0.5 |  |
| Micafungin (2) |  |  |  |  |  | 0 0.0 | 1 50.0 | 0 50.0 | 0 50.0 | 1 100.0 |  |  |  |  |  |  | 0.06 |  |
| Itraconazole (2) |  |  |  |  |  |  |  |  |  |  |  |  |  | 0 0.0 | 2 100.0 |  | >8 |  |
| Amphotericin B (2) |  |  |  |  |  |  |  |  |  |  |  | 0 0.0 | 1 50.0 |  | 1 100.0 |  | 4 |  |
|  | | | | | | | | | | | | | | | | | | |
| *Scopulariopsis brevicaulis/Scopulariopsis brumptii* | | | | | | | | | | | | | | | | | |  |
| Manogepix (1) |  |  | 0 0.0 | 1 100.0 |  |  |  |  |  |  |  |  |  |  |  |  |  |  |
| Voriconazole (1) |  |  |  |  |  |  |  |  |  |  |  |  |  | 0 0.0 | 1 100.0 |  |  |  |
| Anidulafungin (1) |  |  |  |  |  |  |  |  |  |  |  |  | 0 0.0 |  | 1 100.0 |  |  |  |
| Micafungin (1) |  |  |  |  |  |  |  |  |  | 0 0.0 | 1 100.0 |  |  |  |  |  |  |  |
| Itraconazole (1) |  |  |  |  |  |  |  |  |  |  |  |  |  | 0 0.0 | 1 100.0 |  |  |  |
| Amphotericin B (1) |  |  |  |  |  |  |  |  |  |  |  |  | 0 0.0 |  | 1 100.0 |  |  |  |
|  | | | | | | | | | | | | | | | | | | |
| *Trichoderma longibrachiatum* | | | | | | | | | | | | | | | | | |  |
| Manogepix (1) |  |  |  |  |  | 0 0.0 | 1 100.0 |  |  |  |  |  |  |  |  |  |  |  |
| Voriconazole (1) |  |  |  |  |  |  |  |  |  | 0 0.0 | 1 100.0 |  |  |  |  |  |  |  |
| Anidulafungin (1) |  |  | 0 0.0 | 1 100.0 |  |  |  |  |  |  |  |  |  |  |  |  |  |  |
| Micafungin (1) |  |  |  | 0 0.0 | 1 100.0 |  |  |  |  |  |  |  |  |  |  |  |  |  |
| Itraconazole (1) |  |  |  |  |  |  |  |  |  |  |  |  |  | 0 0.0 | 1 100.0 |  |  |  |
| Amphotericin B (1) |  |  |  |  |  |  |  |  |  |  |  | 0 0.0 | 1 100.0 |  |  |  |  |  |
|  | | | | | | | | | | | | | | | | | | |
| *Verruconis gallopava* | | | | | | | | | | | | | | | | | |  |
| Manogepix (2) |  |  |  |  |  |  |  |  | 0 0.0 | 1 50.0 | 1 100.0 |  |  |  |  |  | 0.5 |  |
| Voriconazole (2) |  |  |  |  |  |  |  | 0 0.0 | 1 50.0 | 0 50.0 | 0 50.0 | 0 50.0 | 1 100.0 |  |  |  | 0.25 |  |
| Anidulafungin (2) |  |  |  |  |  | 0 0.0 | 1 50.0 | 1 100.0 |  |  |  |  |  |  |  |  | 0.06 |  |
| Micafungin (2) |  |  |  | 0 0.0 | 2 100.0 |  |  |  |  |  |  |  |  |  |  |  | 0.015 |  |
| Itraconazole (2) |  |  |  |  |  |  | 0 0.0 | 1 50.0 | 0 50.0 | 1 100.0 |  |  |  |  |  |  | 0.12 |  |
| Amphotericin B (2) |  |  |  |  |  |  | 0 0.0 | 1 50.0 | 0 50.0 | 1 100.0 |  |  |  |  |  |  | 0.12 |  |

^a^ Greater than the highest concentration tested.
